# Drone flight data reveal energy and greenhouse gas emissions savings for very small package delivery

**DOI:** 10.1016/j.patter.2022.100569

**Published:** 2022-08-05

**Authors:** Thiago A. Rodrigues, Jay Patrikar, Natalia L. Oliveira, H. Scott Matthews, Sebastian Scherer, Constantine Samaras

**Affiliations:** 1 Department of Civil and Environmental Engineering, Carnegie Mellon University, 5000 Forbes Avenue, Pittsburgh, PA 15213, USA; 2 Robotics Institute, Carnegie Mellon University, 5000 Forbes Avenue, Pittsburgh, PA 15213, USA; 3 Department of Statistics and Data Science, Carnegie Mellon University, 5000 Forbes Avenue, Pittsburgh, PA 15213, USA; 4 Machine Learning Department, Carnegie Mellon University, 5000 Forbes Avenue, Pittsburgh, PA 15213, USA

**Keywords:** quadcopter drone, last-mile delivery, energy consumption, greenhouse gas emissions, robot delivery, autonomous delivery

## Abstract

Uncrewed aerial vehicles (UAVs) for last-mile deliveries will affect the energy productivity of delivery and require new methods to understand energy consumption and greenhouse gas (GHG) emissions. We combine empirical testing of 188 quadcopter flights across a range of speeds with a first-principles analysis to develop a usable energy model and a machine-learning algorithm to assess energy across takeoff, cruise, and landing. Our model shows that an electric quadcopter drone with a very small package (0.5 kg) would consume approximately 0.08 MJ/km and result in 70 g of CO_2_e per package in the United States. We compare drone delivery with other vehicles and show that energy per package delivered by drones (0.33 MJ/package) can be up to 94% lower than conventional transportation modes, with only electric cargo bicycles providing lower GHGs/package. Our open model and coefficients can assist stakeholders in understanding and improving the sustainability of small package delivery.

## Highlights

• Drones can have up to 94% lower energy consumption per package than other vehicles
• Small quadcopter drones consume approximately 0.08 MJ/km and emit 70 gCO_2_e/package
• Total energy consumption can be reduced by 34% by reducing vertical takeoff
• GHG emissions are up to 93% higher in regions with a more carbon-intensive grid mix

## The bigger picture

The growing demand for fast, contactless deliveries has been driving firms to experiment with automated package-delivery vehicles that can avoid urban traffic and rapidly reach customers. Widespread adoption of uncrewed aerial vehicles (UAVs) to replace a portion of first/last-mile truck pickups and deliveries could reshape transportation energy by changing demand patterns and shifting fuel use from fossil fuels to electricity. There is a need to optimize drones and systems to maximize energy productivity. Here, we help stakeholders and researchers understand the energy use of package-delivery drones and provide an open model based on 188 delivery-drone flights with a range of payloads, speeds, and altitudes. We show the impact of the cruise speed and payload mass on the drone’s range, provide generalizable coefficients for others to estimate drone energy use, and show that the energy per package delivered by drones can be up to 94% lower than conventional transportation modes.

## Graphical abstract

**Figure:**
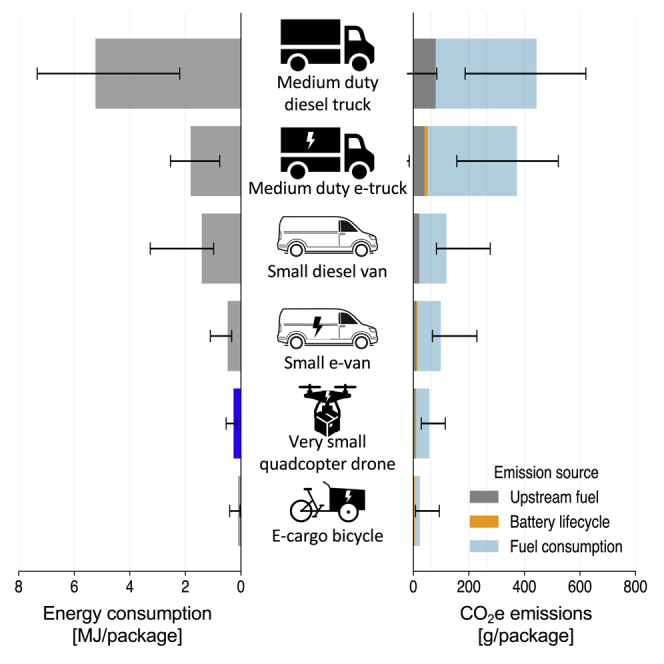

## Teaser

We help stakeholders and researchers understand the energy use of package-delivery drones and provide an open model based on 188 delivery-drone flights with a range of payloads, speeds, and altitudes. We show the impact of the cruise speed and payload mass on the drone’s range, provide generalizable coefficients for others to estimate drone energy use, and show that energy per package delivered by drones can be up to 94% lower than conventional transportation modes.

## Introduction

Achieving large improvements in the energy productivity of freight transportation is challenging, especially in the overwhelmingly petroleum-powered transport sector where medium and heavy trucks in the United States comprise 24% of transportation energy use. This sector is responsible for 37% of transportation-related greenhouse gas (GHG) emissions, while light-duty vehicles comprise 57% of transportation GHG emissions and 64% of transportation energy use. In addition, transportation remains a large source of nitrogen oxides (NOxs) and other air pollutants.^1^ However, the way that consumers are obtaining goods in the United States is changing rapidly.^2^

Even before COVID-19, the growing demand^3^ for fast, contactless deliveries has been driving firms to experiment with automated package-delivery vehicles, such as uncrewed aerial vehicles (UAVs), that can avoid traffic in urban centers.^4^^,^^5^ Initial survey data of 483 customers in Portland, Oregon by Pani et al.^6^ show that COVID-19 is contributing to an environment where more than 60% of online customers are willing to pay extra to receive their packages using autonomous delivery robots. Nevertheless, along with technology and policy challenges, increased shipping costs is a limitation for the adoption of autonomous delivery vehicles.^7^

The appeal of delivery robots also reflects new physical distancing demands to avoid the spread of coronavirus in product deliveries,^8^ and as autonomous delivery technologies advance, new companies emerge to compete for this market niche.^9^ At the same time, alternative transport modes, such as electric cargo bicycles, are becoming cost-effective alternatives to delivery trucks for short-distance deliveries,^10^ drastically reducing the CO_2_ emissions of last-mile delivery in dense metropolitan areas.^11^ With the increased electrification of delivery vehicles, the energy consumption and environmental impacts of the transportation sector are expected to change drastically over the coming years,^12^^,^^13^ and technology, policy, and demand are primary drivers. Widespread adoption of UAVs to replace a portion of first/last-mile truck pickups and deliveries could reshape this sector by changing demand patterns and shifting fuel demands from fossil fuels to electricity. Autonomous delivery robots are coming to the transportation sector, but how these vehicles and systems could be designed to maximize energy productivity is less clear.

So far, a few studies have estimated the energy consumption of quadcopter vehicles, and the energy estimations vary considerably among the different methods used.^14^ Some studies have created models based on theoretical principles,^15^, ^16^, ^17^, ^18^, ^19^, ^20^, ^21^, ^22^, ^23^ while others have developed models based on regression models built on small flight samples.^17^^,^^21^^,^^24^ Finally, a comparison of the energy consumption and GHG emissions between package-delivery UAVs and different transportation modes have been estimated by a few studies,^20^^,^^25^, ^26^, ^27^ but alternative emerging delivery modes, such as electric cargo bicycles, are not included. Our study builds a model based on a much larger empirical test sample of a drone operating at altitudes between 25 and 100 m and with drone ground speeds that vary from 4 to 12 m/s in flights operating under wind conditions varying from 2 to 16 knots. We show that, within these limits, the ground speed and the wind condition impact little on the average energy consumption of the quadcopter drone and that the induced power at a hover and no-wind condition can be used as a good estimator of the average power experienced by the aircraft throughout the flight.

The adoption of a multirotor UAV was motivated by the commercial use of similar aircraft by last-mile delivery companies. For example, the company SpeedBird Aero has used a multirotor with a capacity of 2 kg to deliver food in Latin America.^28^ The Irish drone startup Manna^29^ uses a quadcopter drone to carry payloads varying between 2 and 4 kg.^30^ The DJI Matrice 100 (M100) used in this study is a smaller quadcopter drone and was tested with a maximum payload of 0.5 kg. The M100 carrying a very small payload is likely smaller than purpose-built package-delivery drones for larger packages, which require future work to understand specific energy characteristics. However, we believe that the results using the M100 can provide important information to researchers and industry professionals working with UAVs for last-mile deliveries for very small packages such as medical deliveries, critical parts, or other time-sensitive payloads. Here, we help stakeholders and researchers understand the energy use of uncrewed aerial package-delivery drones. We provide an energy model based on extensive empirical data from 188 flights of a quadcopter drone M100, from which we developed a high-resolution dataset of package-delivery-drone energy use.^31^ In addition, we develop an algorithm that automatically identifies the flight regime across takeoff, cruise, and landing. We show the impact of the cruise speed and payload mass on the drone’s range and provide energy-use coefficients. We use our energy model to compare the energy consumption and GHG emissions of the drone with delivery trucks, delivery vans, and electric cargo bicycles on a distance and package basis. We show how the drone’s emissions differ regionally in the US according to the electricity mix. We perform a sensitivity analysis on the energy consumption on a distance basis (MJ/km) and show the minimum drone energy consumption required to match different vehicles, which can help inform drone designers for future efficient UAVs. Finally, we show the delivery intensity required to match the GHG emissions of the drone for each region of the US.

## Results

We conducted a first-principles analysis and developed a model to estimate the energy required to power a quadcopter. Each of the flight regimes (takeoff, cruise, and landing) were modeled separately, so each energy model was treated as a model class and three different optimal models from that class were selected, one per regime. In order to fairly compare the model classes’ performance and avoid overfitting, we split the data into train and test folds following a stratification strategy by flight ID number. With 120 flights, the training fold was used to estimate the parameters of each model, which were then applied to the remaining 68 flights from the test fold in order to evaluate the performance of the energy models on unseen data.

### Energy model derived from flights

Our energy (E) model uses the induced power $(P_{i}$), which is the power required to overcome gravity in a hover-no-wind situation, as a parameter estimator of the average power observed throughout the flight.

(Equation 1) $$E=\left(b_{1}^{\text{′}}P_{i}+b_{0}\right)t\text{,}$$

where t is the flight duration, and $b_{1}^{\prime }$ and $b_{0}$ are coefficients that linearly correlate $P_{i}$ and the average power throughout the flight.

The *P*_*i*_, used in Equation 1, is calculated as

(Equation 2) $$P_{i}=\frac{{\left(mg\right)}^{1.5}}{\sqrt{2\rho A}}\text{,}$$

where m is the total mass of the drone (including the payload), g is the acceleration of gravity, ρ is the air density that we obtained from the closest airport station (KAGC), and A is the total area under the propellers. Our experiment focused on one type of quadcopter drone; hence, A is constant here.

Combining Equation 2 and Equation 1,

(Equation 3) $$E=\left(b_{1}\frac{m^{1.5}}{\sqrt{\rho }}+b_{0}\right)t\text{,}$$

where $b_{1}$ includes the constants A and g. The estimated coefficients and their standard errors are shown in Table 1.

**Table 1:** Model coefficient ± bootstrap standard error

Coef.	Takeoff	Cruise	Landing
b_1_	80.4 ± 2.6	68.9 ± 2.0	71.5 ± 1.7
b_0_	13.8 ± 18.9	16.8 ± 15.0	−24.3 ± 12.5
R^2^	0.84	0.85	0.90

The coefficients shown in Table 1 were obtained by performing a linear regression between $P_{i}$ and the average power observed throughout each of the 120 flights. The results were then applied to the remaining flights, and the absolute relative error was 2.1% on average, proving the accuracy of the energy model in terms of estimation of energy consumption.

**Figure 1:**
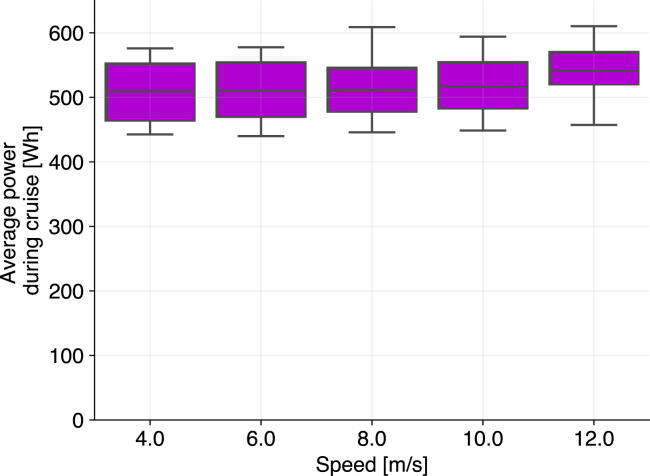
Our data show that for the range of cruise speeds tested, there is no practical variation in the drone’s average power consumption (approximately 5% variation between the median of flights at 4 and 12 m/s)

With the energy model validated, we estimated the energy consumption of a package delivered by a small quadcopter drone. Our energy model suggests that within the speed range tested, the average power consumption of a quadcopter does not vary considerably with the speed during cruise, which has also been observed with our dataset (Figure 1). The speed does affect total flight time, and hence for the drone and range of speeds we tested, total power consumption of each delivery will be higher when flight speeds are lower.

### Drone energy use and GHG emissions

**Figure 2:**
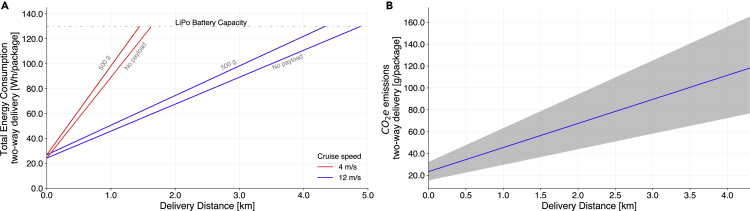
Small drone total energy consumption and greenhouse gas emissions by distance (A) Total energy consumption by distance of delivery varying payload mass and cruise speed. Total trip energy is higher at lower speeds for a fixed distance. (B) CO_2_e emissions of delivering a payload of 0.5 kg at a cruise speed of 12 m/s, the fastest speed evaluated, based on the delivery distance. The uncertainty area corresponds to US regional grid emissions factors. The total energy and CO_2_e correspond to takeoff, cruise from the origin to destination, and landing loaded and takeoff, cruise from destination to origin, and landing empty. As an energy limitation, the nominal capacity of an LiPO TB48D battery is 130 Wh. Results shown here have an altitude during cruise of 100 m, takeoff speed of 2.5 m/s, and landing speed of 2 m/s.

The GHG emissions of a package delivery by drone will depend on the total electricity needed for the delivery and the emissions intensity of the regional electricity grid.^20^ Our analysis shows that variations in the cruise speed have a large impact on the total energy consumption per trip and, consequently, the range of the drone. Because the total time of flight is reduced as the speed increases, a faster speed (with normal operating parameters) for a quadcopter generally enables longer delivery distances for the same amount of energy. We show the influence of speed, payload, and delivery distance on total energy consumption in Figure 2A.

We then calculated the GHG emissions per package delivered based on the US regional non-baseload electricity GHG emissions from the US Environmental Protection Agency,^32^ upstream electricity generation emissions, and battery life cycle emissions. In Figure 2B, we show the GHG emissions per package delivery as a function of the delivery distance. We show the results for the fastest speed evaluated, 12 m/s; however, the model enables evaluation across the full range of speeds, altitudes, and payloads.

**Figure 3:**
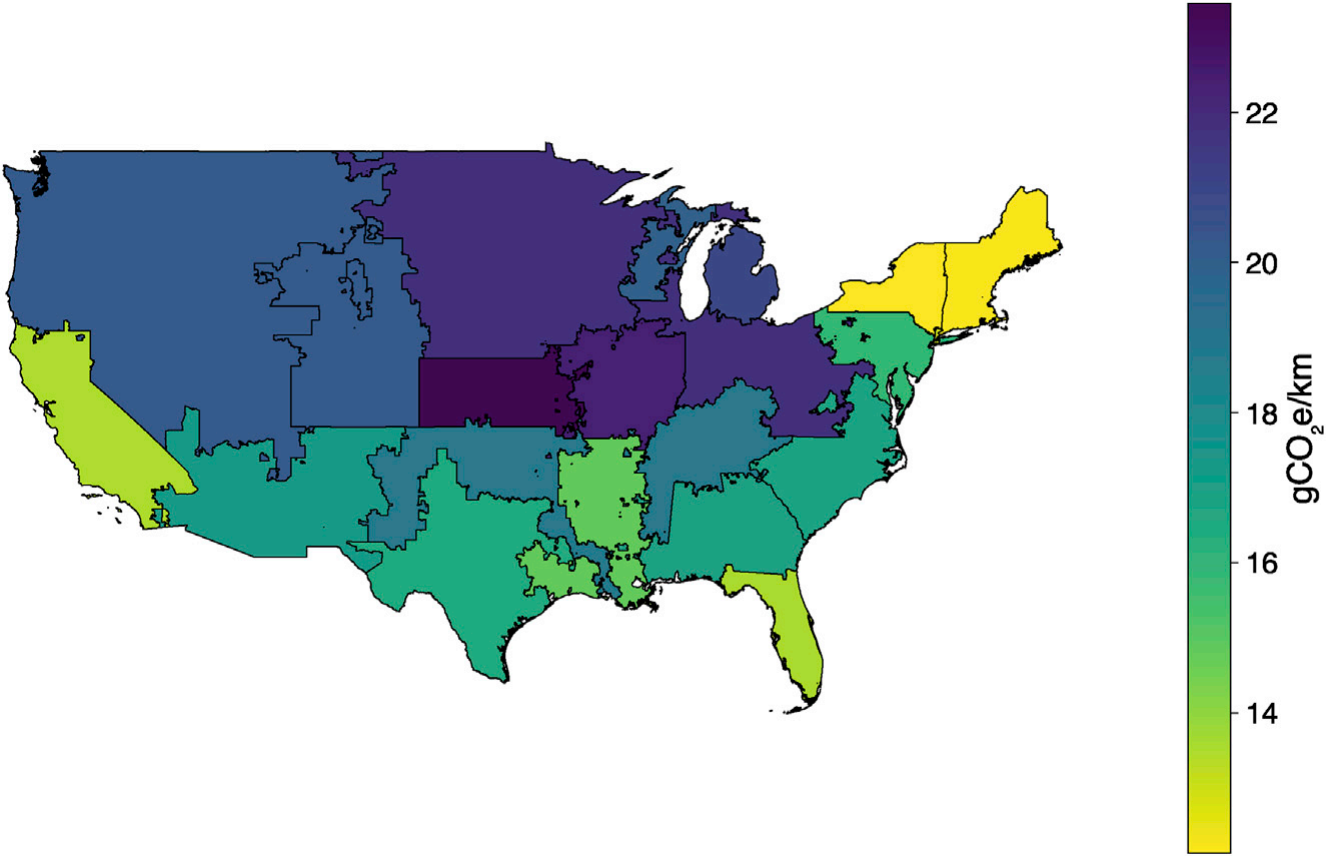
GHG emissions per km from a drone package delivery with a 0.5-kg payload and 2-km one-way delivery distance (4-km round trip), according to the sub-region’s non-baseload electricity grid carbon intensity

We illustrate the impacts of the GHG intensity of a regional electricity grid on drone package delivery GHGs in Figure 3. Using regional non-baseload emissions factors, we show that a drone package delivery in the carbon-intensive central Midwest would emit up to 93% more CO_2_e per km traveled (23.5 g CO_2_e/km) compared with regions with cleaner grid mixes such as New York, which would result in drone emissions of 12.1 g CO_2_e/km.

### Comparison between different transportation modes

We compared the energy consumption of quadcopter drones against diesel and electric medium-duty trucks and small vans and electric cargo bicycles.

**Figure 4:**
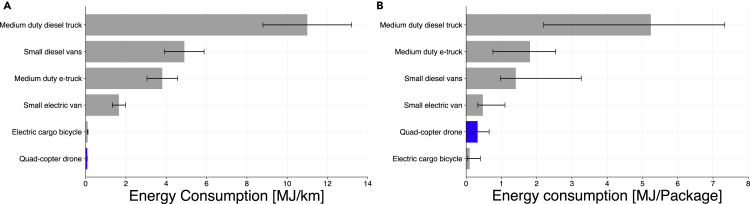
Energy consumption per distance and per package delivered for different transportation modes Error bars represent variations in (A) driving styles and vehicle characteristics and (B) number of packages delivered per distance.

The total energy consumption per distance of small quadcopter drones is among the lowest across transportation modes, as the vehicle is small, light, and has lower payload capacity (Figure 4A). Figure 4B shows the energy consumption per package of drone-equivalent deliveries, i.e., assuming that all packages delivered by the other modes are within the payload and space capacity of a small quadcopter drone. On an energy consumption per package basis, small quadcopter drones and electric cargo bicycles are among the most energy-efficient modes for small package delivery. The number of stops per kilometer and the number of packages delivered per stop varies according to the transportation mode and delivery density (highly dense areas are more likely to have more stops and packages delivered per kilometer).

**Figure 5:**
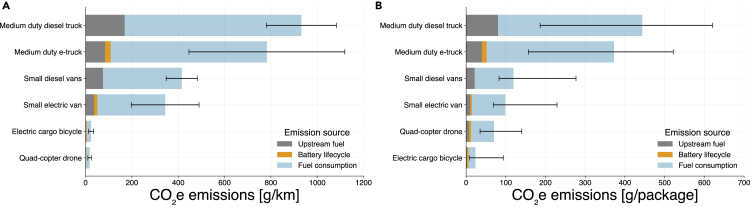
GHG emissions per distance and per package delivered for different transportation modes Error bars (A and B) represent uncertainties due to variations on fuel carbon intensity, battery life cycle emissions, and number of packages delivered per distance.

Similarly, an analysis of the GHG emissions of the fuel of each transportation mode shows that quadcopter drones and electric cargo bicycles are among the most efficient vehicles in g of CO_2_e per km (Figure 5A) and a competitive alternative in terms of GHG emissions per package (Figure 5B). On the other hand, it is important to note that small drones are considerably limited in terms of weight and volume of the packages transported. Therefore, an analysis of the energy consumption and GHG emissions on a per metric ton-km basis in Figure S1 shows that small drones are the most energy-intensive vehicles. Also, local airspace regulations, such as not flying over people and/or motor vehicles,^33^ could impose longer delivery routes, were not considered in this study, and could potentially increase the drone’s energy consumption and GHG emissions per package delivered.^34^

We compared our results with values provided by the United Parcel Service (UPS). In 2019, UPS reported the energy intensity for US Domestic Package operations was 28 MJ/package, from which ground vehicles represented approximately 9.5 MJ/package or 34% (airline fuel, facility heating fuel, and indirect energy correspond to 60%, 3%, and 3%, respectively), with GHG emissions (CO_2_e) intensity of 1 kg/package.^35^ It is important to note that these values encompass the entire ground fleet rather than only last-mile delivery.

Finally, assuming a base case where the drone delivers an average of 0.25 packages per km (one-way delivery distance of 2 km) and consumes 0.08 MJ/km, we calculated the minimum number of packages per km required by each vehicle to match the drone’s energy consumption and CO_2_e per package. Table 2 shows that a medium-duty diesel truck would require approximately 34 packages per km to meet the drone’s performance, which would correspond to having 200 packages delivered in a route of less than 6 km. On the other hand, a small electric van would require a delivery intensity of approximately five packages per km, or a 39-km route to deliver 200 packages, which could potentially be achieved in dense urban centers.

**Table 2:** Delivery density of each mode to match the drone’s energy consumption per package, and the minimum drone energy consumption to match the vehicle’s energy consumption per package

Vehicle	Delivery density required to match drone energy consumption (package/km) (multiplier from base case)	Minimum energy consumption required for the drone (MJ/km)
Medium-duty diesel truck	33.8 (16.1×)	1.31
Small diesel van	15.0 (4.3×)	0.35
Medium-duty electric truck	11.7 (5.6×)	0.45
Small electric van	5.1 (1.5×)	0.12
Electric cargo bicycle	0.3 (0.3×)	0.03
Small quadcopter drone (base case)	0.25	0.07

Moreover, Table 2 also shows the minimum energy consumption required for the drone to match each vehicle’s energy consumption per package. To have a similar energy consumption per package delivered by a medium-duty diesel truck, a small quadcopter drone would need to consume 1.31 MJ/km (approximately 19 times more than our base-case estimate). Figure S2 provides a similar analysis considering that the GHG emissions showing that a diesel truck would need to deliver between 10 and 19 packages per km depending on the region of operation. Future work could estimate of the density of deliveries that would support operations with similar delivery density on a per-area basis to understand which communities are likely to have energy and GHG savings with drone deliveries.

## Discussion

A small quadcopter drone, with a payload of 0.5 kg operating at a cruise speed of 12 m/s and a cruise altitude of 100 m, consumes approximately 0.08 MJ/km and generates 70.1 g of CO_2_e per package when charged on average non-baseload US electricity, with a range of 48.5 g per package in New York (the cleanest US electricity region) to 93.8 g per package in the most carbon-intensive region in the central Midwest. Only electric cargo bicycles had a lower carbon footprint per package, from 16.8 g per package in New York, 23.4 g per package for the average US, and 30.8 g per package in the central Midwest. As the electricity grid gets cleaner over time, the carbon intensity of delivery with electricity-powered vehicles, whether drone, cargo bicycle, van, or truck, will continue to improve.

Our energy model has simple and accurate coefficients that can provide stakeholders and researchers with a drone-energy-consumption estimation for speeds below 12 m/s for similar small drones. However, at greater speeds or using drones with more surface area or mass, a more comprehensive energy-profile method could provide more accurate predictions.

The energy consumption of a very small, commercially available quadcopter drone with payload of 0.5 kg is comparable to the most energy-efficient modes of last-mile delivery when the total mass of delivery is not the main feature considered. For example, in delivery situations where small and light items with high added value, such as medical deliveries, critical packages, and small electronics, very small drones might become a competitive tool to reduce transportation emissions in large urban centers.^36^ In these scenarios, we found that drones can reduce the energy consumption by 94% and 31% and GHG emissions by 84% and 29% per package delivered by replacing diesel trucks and electric vans, respectively. We also found that electric cargo bicycles had similar or lower GHGs per package than drones. We also found that the delivery intensity, i.e., the number of packages delivered per km, and the fuel carbon intensity are the main factors contributing to the drone’s comparative energy and environmental performance.

Adding empirical tests to assess the impacts of varying the area of the propeller, the drone geometry and delivery intensity that were not included in this study could provide an important contribution to this field in future work. It is also important to note that the drone used to collect the data was not optimized to minimize energy consumption, which could further improve its efficiency. The drone used was designed for a very small package, and future studies should include heavier payloads. Given the potential for improved energy productivity of delivery and reduced GHGs per package, very small package delivery by drone and electric cargo bicycles can play an important role in reducing the energy and climate impacts of package delivery.

## Experimental procedures

### Resource availability

#### Lead contact

Further information should be directed to and will be fulfilled by the lead contact, Dr. Samaras (csamaras@cmu.edu).

#### Materials availability

This study did not generate new unique reagents.

### Empirical data collection from drone flight

We performed a series of flights to empirically measure the energy consumption of a quadcopter UAV. An experimental protocol was created and followed to ensure a reliable approach for data acquisition.^31^

An M100 quadcopter was equipped with an anemometer, current and voltage monitor, GPS, and accelerometer collecting data on wind speed and direction, battery current and voltage demand, and position, orientation, velocity, and acceleration. The flights were performed in a pre-established route with varying altitudes (25, 50, 75, and 100 m), speeds (4, 6, 8, 10, and 12 m/s), and payload mass (no payload, 250 g, and 500 g). The sensors and computer box attached to the drone weighed approximately 1,200 g. Each combination was repeated at least three times, totaling 188 flights. The data provided by each sensor were synchronized to a frequency of approximately 5 Hz using the ApproximateTime^38^ message filter policy of Robot Operating System (ROS).

For a better understanding of the energy-consumption profile of each flight, we created an algorithm to automatically divide the data into three different flight regimes: takeoff, cruise, and landing (Figures S3–S6). The data available in Rodrigues et al.^37^ were processed using this algorithm before the analysis described in the next sections.

### First-principles analysis

The energy required to power a UAV can be estimated using a first-principles analysis based on helicopter aerodynamics.^39^ First, we defined the working coordinate frames for a quadcopter drone (Figures S7–S9). Then, we assessed the power required to maintain the drone at a steady hover condition. Finally, we expanded the power analysis to include other power demands.

**Figure 6:**
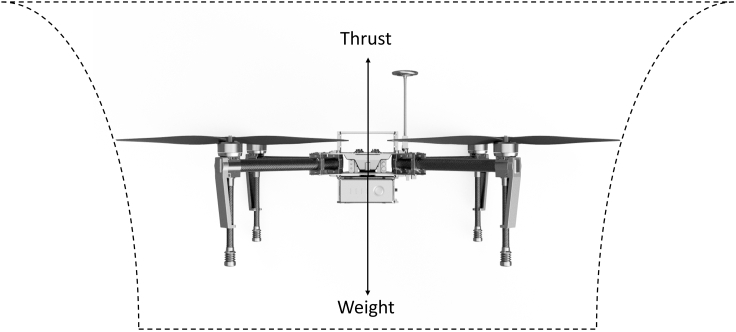
Drone flying at a hover and no-wind condition

The main power demand of a drone is in the form of $P_{i}$. The *P*_*i*_ represents the power required to overcome the force of gravity in order to keep the aircraft in the air, and it can vary according to the flight maneuver.^39^ The most basic way to estimate $P_{i}$ is considering a hover condition without wind (Figure 6).

In that case, the thrust (T) equals the only force acting on the drone: its weight (*W* = *mg*),^39^ and $P_{i}$ can be estimated as

(Equation 4) $$P_{i}=Tv_{i}\text{,}$$

where $v_{i}$ is the induced velocity.

During hover, $v_{i}$ can be simplified as

(Equation 5) $$v_{i}=\sqrt{\frac{T}{2\rho A}}\text{,}$$

where ρ is the air density and A is the total area covered by all four propellers.

Combining Equations 5 and 4

(Equation 6) $$P_{i}=\frac{{\left(T\right)}^{3/2}}{\sqrt{2\rho A}}=\frac{{\left(mg\right)}^{1.5}}{\sqrt{2\rho A}}\text{,}$$

where m is the total mass of the drone and g is the gravitational acceleration.

More details of the first-principles analysis and an expanded first-principles energy model are available in the supplemental information (Figures S10 and S11).

### Regression-based energy model

Our energy model inquires how effectively $P_{i}$ can be used as an estimator for the energy consumed during a package-delivery flight. In such a case, the average power ($\bar{P}$) throughout the flight is modeled as a linear regression of the *P*_*i*_

(Equation 7) $$\bar{P}=b_{1}^{\prime }P_{i}+b_{0}\text{,}$$

where $b_{1}$ and $b_{0}$ are the slope and intercept of the linear regression, respectively.

**Figure 7:**
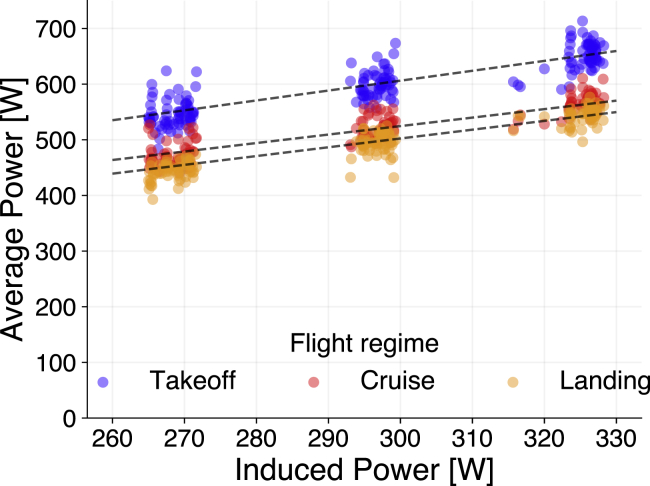
Relationship of induced power to drone average power of flights separated by flight regime Payloads of 0 (left), 250 (center), and 500 g (right). $R^{2}$ are 0.84, 0.85, and 0.90 for takeoff, cruise, and landing, respectively.

We can expand Equation 7 to account for the sum of the three flight regimes (Figure 7) and combine the area of the propeller (A) and gravity acceleration (*g*) from Equation 2 with coefficient $b_{1}$. Thus, the total energy consumption (E) is estimated as

(Equation 8) $$E=\underset{r\in R,l\in L}{\sum }\left(b_{1}^{\left(r,l\right)}{\frac{m^{1.5}}{\sqrt{\rho }}}^{\left(l\right)}+b_{0}^{\left(r,l\right)}\right)t^{\left(r,l\right)}$$

for R={takeoff,cruise,landing} and L={loaded,unloaded}.

The step-by-step calculation used to compute $b_{0}$, $b_{1}$, and $R^{2}$ can be found in the supplemental information under the section linear regression.

### Machine learning for comparison

Evaluating whether the model’s performance is good given the available measurements cannot be inferred from its performance alone. Therefore, we compare the predictive power of the energy model with a flexible nonlinear algorithm,^40^ XGBoost, available in the programming environment R. This boosted tree algorithm prioritizes predictive power against interpretability, and it is appropriate for predictive performance given the available features. If our energy model presents similar accuracy to XGBoost, it indicates that the parametric and functional restrictions we have made for the energy model development are suitable.

We fitted a gradient boosted tree algorithm, XGBoost.^40^ The algorithm was separately trained for each flight regime with a quadratic loss function, and for all regimes, we used 80% as a subsample ratio of both features and observations for each tree. For hyperparameter tuning, we varied learning rate, maximum tree depth, and regularization parameter γ in a grid search approach. 5-fold cross-validation (CV) was used for error estimation; for tuning only, we compared the absolute relative error (ARE; Equation 9) instead of the quadratic error. We fixed a regime and a set of hyperparameter values to be tuned then varied the following hyperparameters: learning rate (0.01, 0.05, 0.1), maximum depth of each weak learner (3, 5, 8), and minimum loss reduction required to make another partition (0, 1, 5). Then, we ran XGBoost on the training data using squared loss as the objective function. Our response variable and features were the same used in the first-principles energy model. We used the stochastic approach by subsampling 80% of the data at each tree fitting. Our algorithm used up to 1,000 trees; however, if there was no significant increase in performance after 50 trees, the algorithm stopped. Then, we plotted the number of trees (up to 1,000) versus training error and estimated test error via 5-fold cross validation.

After tuning, the model was trained with the optimal hyperparameters on the entire training set, and AREs were computed for the flights on the test set. We selected the hyperparameter combination to be used for each regime (number of trees, learning rate, maximum depth, minimum loss reduction) by choosing a low test error, low generalization error, and low standard errors. The selected hyperparameter values are in Table S1.

### Estimation of the standard errors for the coefficients

To obtain standard errors of the estimated coefficients, we used a nonparametric bootstrap approach.^41^ 1,000 bootstrap replications were used to resample with replacement the 120 training flights, and the energy models for the three flight regimes were refitted for each bootstrap sample. At the end, the standard errors (SEs) of the coefficients were obtained from their sampling distribution.

### Model predictive power

To evaluate the model’s predictive power, the regime-specific fitted models were then applied to the testing flights of the test set and were compared by ARE, computed at flight resolution. That is, for each flight from the test set, we computed their $E_{measured}$ integrating power over time and the $E_{estimated}^{m}$ as the sum of the integral of the estimated power over time for the three flight regimes via method m:

(Equation 9) $$ARE\left(m\right)=\left|\frac{E_{measured}-E_{estimated}^{m}}{E_{measured}}\right|$$

for m∈{EnergyModel,XGBoost}.

Figure S12 shows the AREs on the test set for the energy models developed in this study.

### Estimation of drone range

Our analysis also shows the impact of varying operational parameters (speed, altitude, and payload) on the range of the drone. The two-way drone range (*d*) can be calculated considering the cruise speed

(Equation 10) $$E=\sum \left(b_{1}\frac{m^{1.5}}{\sqrt{\rho }}+b_{0}\right)t=\sum E_{\;takeoff}+\sum E_{\;landing}+\sum \left(b_{1}\frac{m^{1.5}}{\sqrt{\rho }}+b_{0}\right)\frac{d}{V_{cr}}\text{.}$$

Expanding for a two-way trip and solving for d

(Equation 11) $$d=\frac{\left[E_{max}-\left(E_{\;takeoff}^{\;l}+E_{\;takeoff}^{\;u}+E_{\;landing}^{\;l}+E_{\;landing}^{\;u}\right)\right]V_{cr}}{b_{1}\left({\frac{m^{1.5}}{\sqrt{\rho }}}^{\;l}+{\frac{m^{1.5}}{\sqrt{\rho }}}^{\;u}\right)+2b_{0}}\text{,}$$

where $E_{max}$ is the energy available in the battery; $E_{takeoff}$ and $E_{landing}$ are the energy consumed during takeoff and landing for delivery (loaded = l) and returning (unloaded = u), respectively; $b_{1}$ and $b_{0}$ the coefficients from Table 1 for cruise; and $V_{cr}$ is the average inertial cruise speed.

The energy during takeoff and landing can be calculated as

(Equation 12) $$E=\left(b_{1}\frac{m^{1.5}}{\sqrt{\rho }}+b_{0}\right)\frac{h}{V}\text{,}$$

where h is the cruise altitude and V is the average speed during takeoff and landing.

For instance, a small quadcopter operating at $V_{cr}$= 12 m/s, payload = 500 g, h = 100 m, takeoff average speed ($V_{tk}$) = 2.5 m/s, and landing average speed $V_{ld}$= 2.0 m/s has a range of approximately 4 km (2 km of one-way delivery range), consuming approximately 74.5 Wh (per round-trip delivery).

Therefore, a quadcopter drone flying under these conditions would consume approximately 0.067 MJ/km, not considering charging and transmission losses. The energy consumption during takeoff and landing for this trip corresponds to approximately 36% of the total energy consumption (26.8 Wh per trip). This share of energy could be reduced by 95% in a 5-m takeoff (to 1.34 Wh per trip), which could be achieved, for instance, if the drone would depart from the top of a building, provided sufficient space for safe operation. This would reduce total trip energy by 34% (74.5–48.95 Wh) or increase the range from 2 to 3 km (4–6 km two-way distance), consuming the same amount of energy. Further analysis of system-wide energy benefits of building-to-building drone travel is an important topic for future research.

### Transport mode comparison

We develop a model comparing the small quadcopter drone with different transportation modes in terms of energy consumption and CO_2_e emissions and validate it against top-down sustainability reports from UPS in 2019. The energy consumption of a medium-duty diesel truck is considered as 11 MJ/km^43^. Whereas a medium-duty electric truck has an energy consumption of 1.4 kWh/mile,^42^ or 3.13 MJ/km, diesel vans operate at 18.4 MPG on average,^43^ or 4.9 MJ/km (conversion factors: one gallon of diesel = 137,381 Btu,^44^ 1 MJ = 947.817 Btu, one mile = 1.60934 km). On the other hand, electric vans operate with an energy consumption of 0.38 kWh/km,^45^ or 1.36 MJ/km. Finally, an electric cargo bicycle operates at 0.023 kWh/km,^46^ or 0.08 MJ/km (conversion factors: 1 kWh = 3.6 MJ). In addition, variations in driving style can vary energy consumption by 40%.^47^

Based on our energy model, a small quadcopter drone consumes approximately 74 Wh in a 2-km delivery distance (4-km total distance), or 0.067 MJ/km, when delivering at maximum capacity (0.5-kg payload with unloaded return) and a cruise speed of 12 m/s. Transmission losses of 6.5% and a charging efficiency of 88%^32^^,^^48^, ^49^, ^50^ were included with the energy consumption of the electric vehicles (Table 3). Table S1 summarizes the nominal energy consumption and also provides the payload capacity of each mode.

**Table 3:** Base-case energy consumption and GHG emissions for different vehicles

Vehicle class	Energy consumption (MJ/km)	Fuel GHG emissions (g/km)	Upstream GHG emissions (g/km)	Battery GHG emissions (g/km)	Energy consumption (MJ/package)	GHG emission (g/package)
Medium-duty diesel truck	11.00	762.8	168.7	–	5.24	443.6
Small diesel van	4.90	339.8	75.2	–	1.41	119.2
Medium-duty electric truck	3.80	674.3	83.7	24.5	1.81	372.6
Small electric van	1.65	293.0	36.4	14.1	0.47	98.7
Electric cargo bicycle	0.10	17.9	2.2	3.3	0.10	23.4
Small quadcopter drone	0.08	14.4	1.8	1.3	0.33	70.1

The electricity CO_2_e emissions were considered to be the 2020 American non-baseload average of 177 g/MJ (638 g/kWh), with the lower bound being 111 g/MJ (399 g/kWh) from New England and the upper limit being 250 g/MJ (900 g/kWh), reflecting non-baseload emissions from the central Midwest.^32^ CO_2_e emissions for diesel combustion was considered as 1.61 × 10^−4^ lb/Btu,^51^ or 69.35 g/MJ. Upstream GHG emissions for average diesel and electricity generation are 15 g/MJ and 22 g/MJ^53^, respectively. The battery life cycle emissions for the drone (assumed to be similar to Li-iron phosphate) were calculated as 0.76 (base case), 0.23 (low case), and 1.52 g/km (high case). Similarly, the electric cargo bicycle has battery life cycle emissions of 1.3 g/km, considering a Li-ion NMC811 battery. For the electric van and electric medium-duty truck, we assumed a battery of Li-ion NMC811, resulting in 14.1 g/km for the van and 24.5 g/km for the truck.^52^ Energy and emissions associated with system changes beyond the vehicle (such as in warehousing^20^) are not included and are important areas for future work.

The energy consumption per package ($E_{pack}$) was calculated as

(Equation 13) $$E_{pack}=\frac{E_{dist}}{S_{freq}\cdot P_{freq}}\text{,}$$

where $E_{dist}$ is the energy consumption per distance unit, $S_{freq}$ is the number of stops to deliver packages per distance unit, and $P_{freq}$ is number of packages delivered per stop on average.

Similarly, the GHG emissions per package ($GHG_{pack}$) is calculated as

(Equation 14) $$GHG_{pack}=\frac{E_{dist}\cdot GHG_{energy}}{S_{freq}\cdot P_{freq}}\text{,}$$

where $GHG_{energy}$ is the mass of CO_2_e per energy unit.

For this study, we used the US National Renewable Energy Laboratory’s Fleet DNA: Commercial Fleet Operating Data^53^ to estimate the number of stops per km of delivery trucks and vans. Therefore, we assumed that trucks and vans have an average of 0.7 and 1.74 stops/km, respectively. In addition, we assumed that trucks and vans would deliver an average of three and two packages per stop, respectively. Similarly, we assumed that an electric cargo bicycle and a small quadcopter drone would experience 1 and 0.25 stops/km, respectively, and deliver one package per stop on average. We also varied these parameters in order to assess their sensitivity in the final outcome. Table S2 provides additional details on the values assumed for delivery intensity. Tables S3 and S4 provide life cycle GHG emissions for vehicle per km and package, respectively, for each US Environmental Protection Agency (EPA) sub-region. Table 3 summarizes the values calculated per vehicle.

## Data Availability

All drone data are available at Figshare: https://doi.org/10.1184/R1/12683453, and all modeling codes are available at Zenodo: https://doi.org/10.5281/zenodo.6726991. We collected data on 188 flights to assess the power profile of a package-delivery drone given a set of operational parameters (payload, altitude, and speed during cruise). The data, available at Rodrigues et al.,^37^ and a data descriptor^31^ provide the details of the experiment. In addition, we have developed an algorithm that separates the data into three different flight regimes: takeoff, cruise, and landing, in order to better understand the energy consumption profile during flight. We then perform a first-principles analysis to develop an energy model to estimate the drone’s energy consumption. Finally, we use the energy model to compare the drone with different transportation models from an energy consumption and GHG emissions basis.

## Supplemental information

Document S1. Supplemental experimental procedures, Figures S1–S13, and Tables S1–S4

Document S2. Article plus supplemental information
