# Supplementary material for: Drone flight data reveal energy and greenhouse gas emissions savings for very small package delivery
Source: Patterns (N Y). 2022 Aug 5;3(8):100569. doi: 10.1016/j.patter.2022.100569 (PMC9403403; doi:10.1016/j.patter.2022.100569)
Supplement: Document S1. Supplemental experimental procedures, Figures S1–S13, and Tables S1–S4 [file mmc1.pdf]

Patterns, Volume *3*

Supplemental information

Drone flight data reveal energy and greenhouse gas emissions savings for very small package delivery

Thiago A. Rodrigues, Jay Patrikar, Natalia L. Oliveira, H. Scott Matthews, Sebastian Scherer, and Constantine Samaras

Supplemental Experimental Procedures

S1. Per ton-km comparison

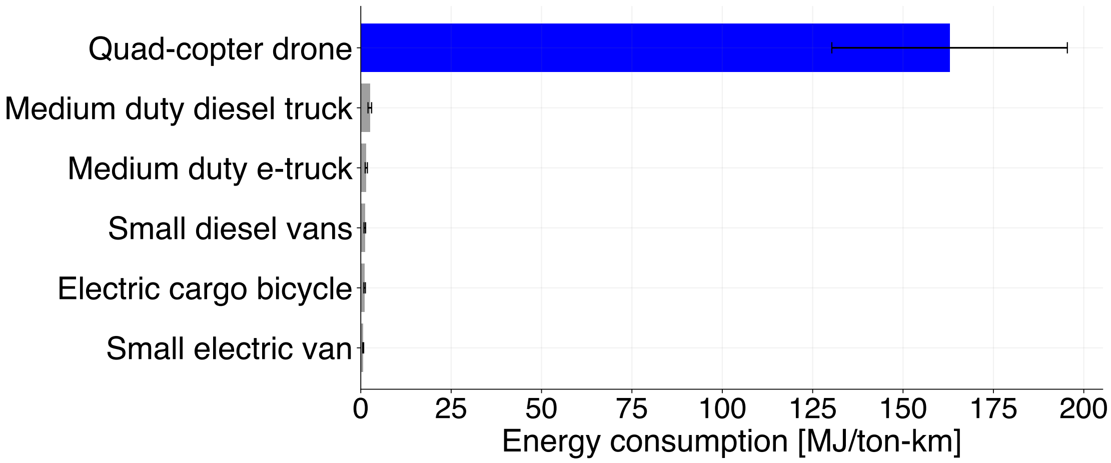

Figure S1: Energy consumption comparison on a per ton-km basis. Error bars represent variations in driving styles and vehicle characteristics

S2. Delivery intensity to match drone's GHG emissions per package

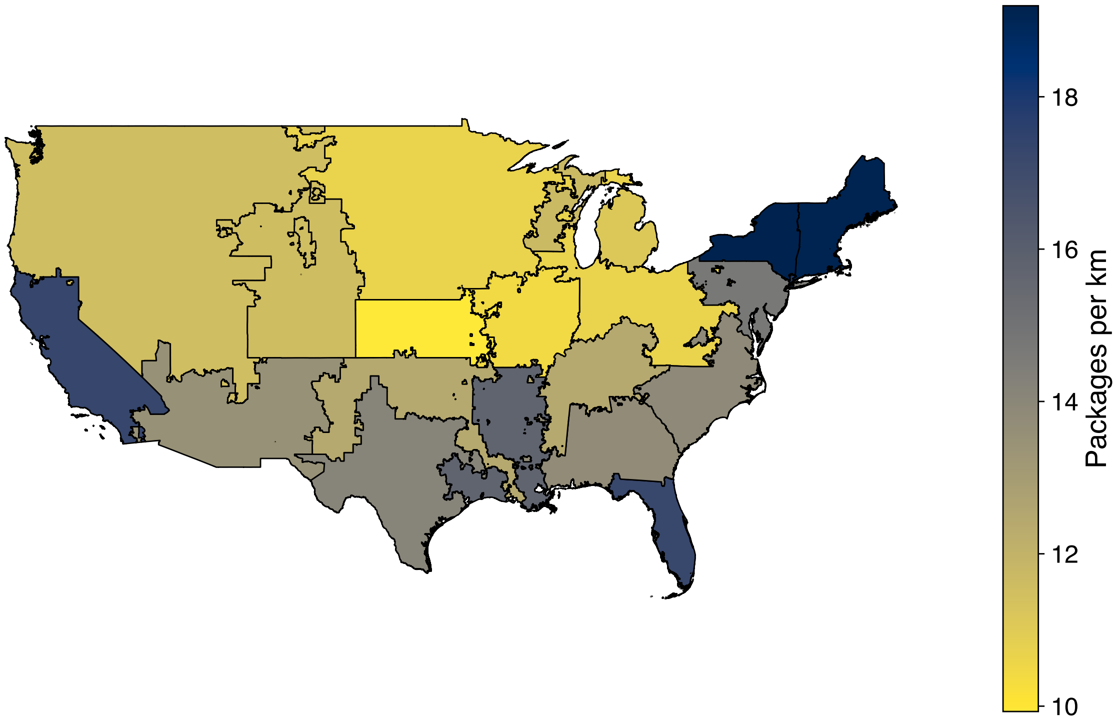

Figure S2: Medium duty diesel truck delivery intensity in packages per km to meet drone's GHG emission per sub-region of the US. Our base model assumes a drone delivering 0.25 packages per km, i.e. a two-way delivery distance of 4 km, consuming 0.27 MJ/package.

S3. Defining flight regimes

An example of the drone's altitude versus time (Figure S3) shows very clearly when the three flight regimes (take off, cruise, and landing) start and end. The steady climbing movement in the first part of the flight rep-

resents the Take Off regime. The relative constant altitude between 50 and 170 seconds correspond to the Cruise regime. Lastly, the steady descent movement at the end of the flight represents the Landing regime.

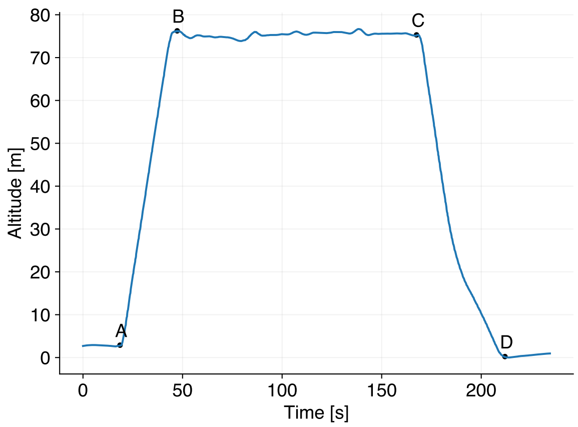

Figure S3: Altitude over time. Points **A**, **B**, **C**, and **D** indicate the starting and ending points for each flight regime. Takeoff, cruise and landing regimes are represented between points **AB**, **BC**, and **CD**, respectively.

Although defining the interval where the three regimes occur seems to be quite intuitive for human eyes, the inherent variability of the data provided by the GPS make the process less efficient for an algorithm. Therefore, a Gaussian Filter was applied to the data in order to reduce eventual noises. A sigma of 5 was used, as it approximately reflects the accuracy of the GPS measurements. The result shown in Figure S4 is a much smoother curve.

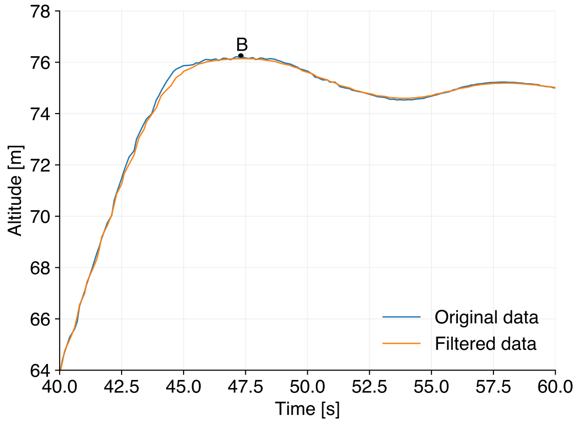

Figure S4: Gaussian filter applied to a portion of the altitude data. Blue curve curve represents the original data and the smoother red curve represents the filtered data. Point **B** indicates the end of the takeoff regime.

Once the Gaussian filter has been applied to the altitude curve, the first derivative provides the points of maximum and minimum altitude variations (Figure S5) that will be used to define the beginning and ending of each regime.

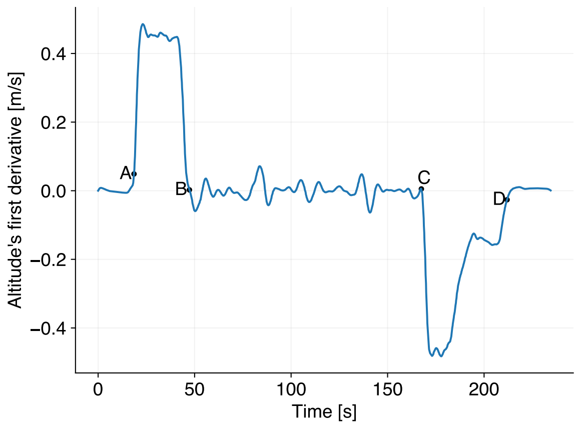

Figure S5: First derivative of the altitude over time. Points **A**, **B**, **C**, and **D** indicate the starting and ending points of the flight regimes. Takeoff, cruise and landing regimes are represented between points **AB**, **BC**, and **CD**, respectively.

It has been established that the Take Off regime would start when the slope reached 0.05 for the first time (**A**) and it would end once the slope reached zero, after reaching maximum slope point (**B**). Similarly, Landing was defined to begin at the first time the slope reached 0 moving backwards from the minimum slope point (**C**) and ends when the slope would reach negative -0.25 moving forward from the minimum slope point (**D**). The Cruise regime is defined as the interval between **B** and **C**.

The result allows the automatic identification of the time interval where the three flight regimes occur throughout the flights. Figure S6 shows the outcome of a particular flight, divided into three regimes.

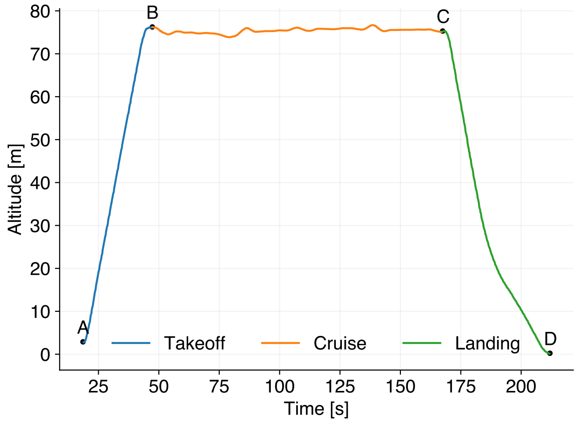

Figure S6: Flight with flight regimes defined. Takeoff, cruise and landing regimes are represented between points **AB**, **BC**, and **CD**, respectively.

The script that automates the flight regime classification is available at https://doi.org/10.5281/zenodo.6726991.

S4. Coordinate Frames

The energy model developed uses a first-principle analysis based on helicopter aerodynamics,[1] which defines different coordinate frames for an aircraft. All the components were considered regarding the drone's body frame as described below.

Different coordinate frames can be used to understand the dynamic behavior of a Unmanned Aerial Vehicle (UAVs).[2] Here we will use the inertial frame, vehicle frame, body frame, stability frame and wind frame, to be defined as follows.

The *inertial frame* has a fixed latitude-longitude origin and uses the North-East coordinates as **i** and **j** directions and the **k** axis towards the center of the Earth (Figure S7a[1]) . Similarly, the *vehicle frame* uses the same north-east-down (NED) directions, but with its origin fixed on the UAV's center of mass (G) (Figure S7b).

[1]CAD Model adapted from[3]

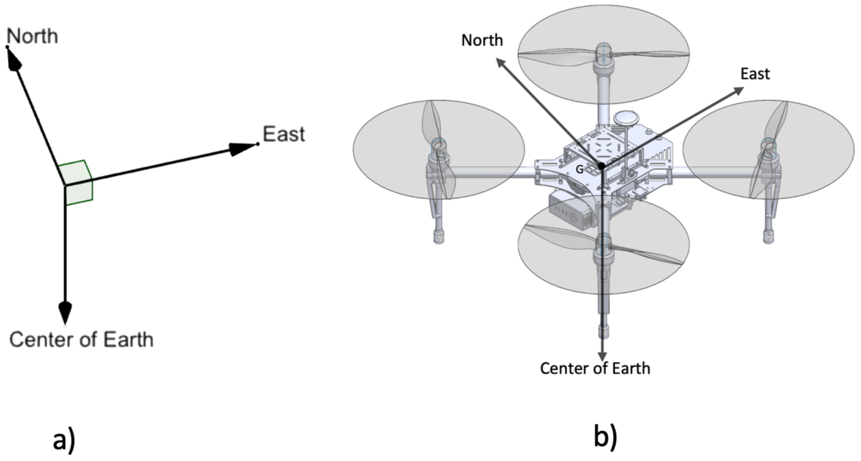

Figure S7: Inertial and Vehicle Frames

The *body frame*, on the other hand, considers the **i**, **j** and **k** directions regarding the body of the aircraft. For instance, the x-axis was defined as the direction along the front of the aircraft and the mass center of the drone (Matrice 100). The angle between the i^v (North) and i^b on the i^vGj^v plane is defined as ψ (Figure S8a). The angle between i^b and i^v on the i^vGk^v plane is defined as θ (Figure S8b). Finally, the angle between j^v and j^b on the j^vGk^v plane is defined as ϕ (Figure S8c).

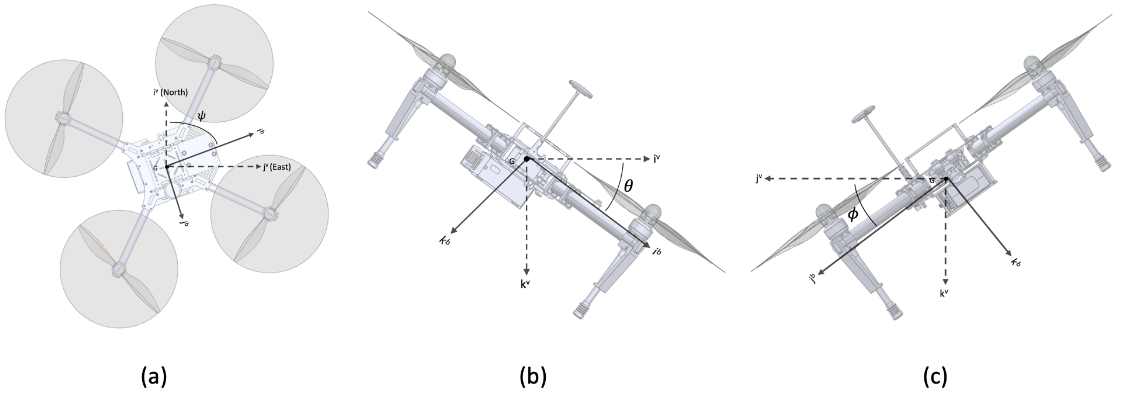

Figure S8: Body Frame

The *stability frame* correspond to the frame of air surrounding the aircraft. The airspeed vector (V_a), defined as the difference between the ground velocity (V_g) and the wind velocity (V_w), flows through the aircraft at a i^sGj^s plane, that is rotated from the body frame reference by α, the angle of attack. Finally, the *wind frame* assumes the **i** axis in the same direction of the airspeed vector. The angle between i^s and V_a is defined as the side slip angle (β) (Figure S9).

With the main working frames described, it is possible to transcribe any vector written as **A** = $a_x\mathbf{i} + a_y\mathbf{j} + a_z\mathbf{k}$ from frame F^0 to frame F^1 using a rotation matrix (R_0^1),

$$\mathbf{A^1} = \left[R_0^1\right]\mathbf{A^0}. \tag{1}$$

For this work, we have established the body frame as the standard working frame. Therefore, all vectors were transcribed to the body frame using the following rotation matrices.[2]

From the vehicle frame to the body frame (R_v^b) the rotation matrix is given as

$$\left[R_v^b\right] = \begin{bmatrix} c_\theta c_\psi & c_\theta s_\psi & -s_\theta \\ s_\phi s_\theta c_\psi - c_\phi s_\psi & s_\phi s_\theta s_\psi + c_\phi c_\psi & s_\phi c_\theta \\ c_\phi s_\theta c_\psi + s_\phi s_\psi & c_\phi s_\theta s_\psi - s_\phi s_\psi & c_\phi c_\theta \end{bmatrix}, \tag{2}$$

where c_θ, c_ψ and c_ϕ are the cosines of the angles θ, ψ and ϕ, respectively, and s_θ, s_ψ and s_ϕ are the sines of the angles θ, ψ and ϕ, respectively.

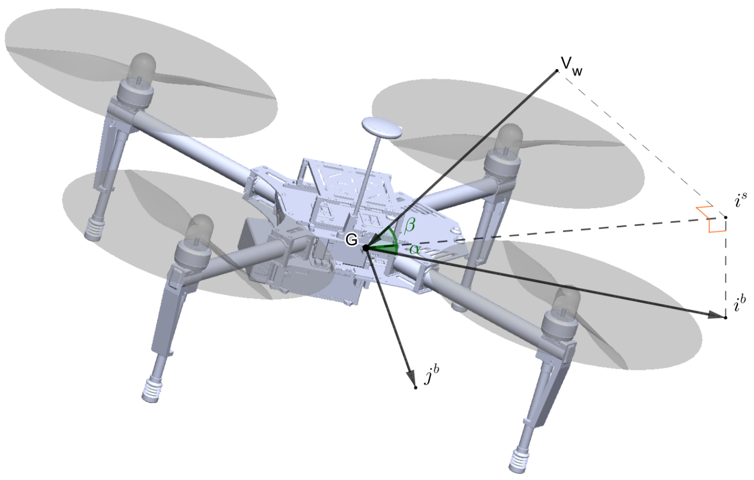

Figure S9: Stability and Wind Frame

The transformation from the stability frame to the body frame is given by the matrix R_s^b

$$[R_s^b] = \begin{bmatrix} \cos(\alpha) & 0 & -\sin(\alpha) \\ 0 & 1 & 0 \\ \sin(\alpha) & 0 & \cos(\alpha) \end{bmatrix}, \tag{3}$$

and the transformation from wind frame to the body frame is given by the matrix R_w^b

$$[R_w^b] = \begin{bmatrix} \cos(\beta)\cos(\alpha) & -\sin(\beta)\cos(\alpha) & -\sin(\alpha) \\ \sin(\beta) & \cos(\beta) & 0 \\ \cos(\beta)\sin(\alpha) & -\sin(\beta)\sin(\alpha) & \cos(\alpha) \end{bmatrix}. \tag{4}$$

S5. First Principles Analysis

The power required for a UAV to maintain a hover in a no-wind condition can be estimated using a first principle analysis established for helicopter aerodynamics. In this case, the power required by a drone can be divided into 4 forms of power: induced power (P_i), profile power (P_p), parasitic power (P_d) and ancillary power (P_a), as described further below.

$$P = P_i + P_p + P_d + P_a \tag{5}$$

S5.1 Induced Power

The induced power (P_i) represents the power required to overcome the force of gravity in order to keep the aircraft in the air, and it can vary according to the flight maneuver.[1] In order to calculate P_i it is necessary to understand how thrust (T) is estimated for a drone propeller. Most of the current models provide thrust from a two-dimension approach, where β = 0 (Figure S10), so that T is given as

$$\mathbf{T} = 2\rho A \mathbf{v_i}\sqrt{(V\cos(\alpha)^2 + (V\sin(\alpha) + v_i)^2}, \tag{6}$$

where ρ represents air density, $\mathbf{v_i}$ induced velocity, V magnitude of the air velocity, and A total area covered by the propellers, calculated by

$$A = M\pi R^2, \tag{7}$$

where M is the number of propellers and R the radius of the propeller.

The induced velocity from a 2D perspective can be estimated through the implicit equation

$$\mathbf{v_i} = \frac{\mathbf{T}}{2\rho A\sqrt{(V\cos(\alpha))^2 + (V\sin(\alpha) + v_i)^2}}. \tag{8}$$

During hover in a no-wind situation, $\alpha = 90°$ and $V = 0$, Equation (8) is reduced to

$$\mathbf{v_i} = \sqrt{\frac{\mathbf{T}}{2\rho A}} \tag{9}$$

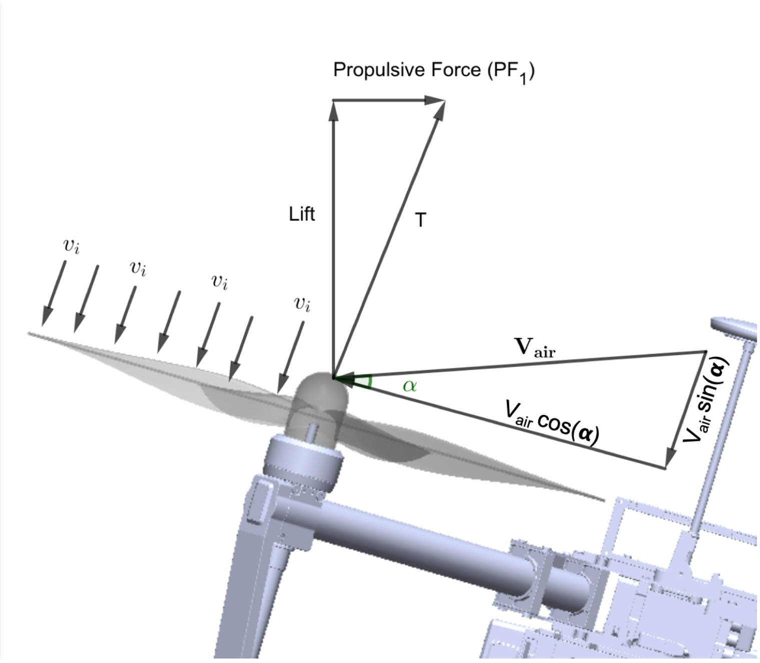

Figure S10: Thrust, β = 0

and $\mathbf{T}$ can be approximated to the drone's weight:

$$T = mg \tag{10}$$

where m represents mass and g gravitational acceleration. Therefore, the induced power during hover in a no-wind condition is calculated as

$$P_i = \mathbf{T} \cdot \mathbf{v_i} = \frac{(mg)^{\frac{3}{2}}}{\sqrt{2\rho A}}. \tag{11}$$

From a spatial perspective **T** must be recalculated to account for $\beta > 0$. Using the same approach used by,[1] and adding β to the relative position of the air flowing through the aircraft, **T** becomes part of a three-dimension force system that accounts for 2 propulsive forces: PF_1 and PF_2 (Figure S11).

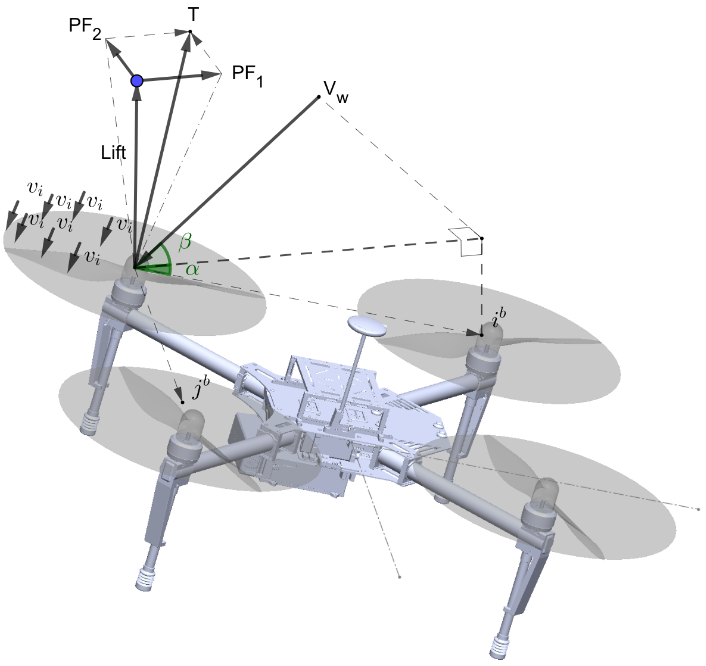

Figure S11: Thrust, $\beta > 0$

$$T = 2\rho A v_i \sqrt{\mathbf{V_i}^2 + \mathbf{V_j}^2 + (\mathbf{V_k} + \mathbf{v_i})^2} \quad (12)$$

where $\mathbf{V_k}$ represents the airspeed vector flowing perpendicularly to the propeller, and $\mathbf{V_i}$ and $\mathbf{V_j}$ are the components acting on the propellers plane. They can be written as a function of $||\mathbf{V}||$ or V, α and β:

$$V_i = V\sqrt{\frac{\sin^2(\alpha)\sin^2(\beta) - \sin^2(\beta)}{\sin^2(\alpha)\sin^2(\beta) - 1}} \quad (13)$$

$$V_j = V\frac{\cos(\alpha)\cos(\beta)}{\sqrt{\sin^2(\alpha)\cos^2(\beta) + \cos^2(\alpha)}} \quad (14)$$

$$V_k = V\sqrt{\frac{\sin^2(\alpha)\sin^2(\beta) - \sin^2(\alpha)}{\sin^2(\alpha)\sin^2(\beta) - 1}} \quad (15)$$

Therefore, P_i for a moving forward flight regime is

$$P_i = T\left(V\sqrt{\frac{\sin^2(\alpha)\sin^2(\beta) - \sin^2(\alpha)}{\sin^2(\alpha)\sin^2(\beta) - 1}} + v_i\right). \quad (16)$$

T can be calculated as a function of θ, ϕ and the rotor lift (L), which in turn can be found as a function of the drone's weight (mg) and the angles θ and ϕ:

$$T = mg\sqrt{\frac{\sin^2(\phi)\cos^2(\theta) + \cos^2(\phi)}{\cos^2(\phi)\cos^2(\theta)}} \quad (17)$$

and from Equation (9), v_i can be calculated through the implicit equation

$$v_i = \frac{\frac{T}{2\rho A}}{\sqrt{V_i^2 + V_j^2 + (V_k + v_i)^2}}. \quad (18)$$

S5.2 Profile Power

The profile power (P_P) represents the power necessary to overcome the drag from the rotating propeller blades.[4] P_p for a hover regime is calculate as

$$P_{p,hover} = \frac{Ncc_{d_{blade}}\rho R^4}{8}\psi\Omega^3 \quad (19)$$

where N is the number of blades in a single propeller, c represents the blade chord width, $c_{d_{blade}}$ is the drag coefficient of the blade, and $\psi\Omega$ is the angular speed.

For a horizontal flight, P_p becomes:

$$P_p = P_{p,hover}(1 + \mu^2) \quad (20)$$

where, μ is the advance ratio for the propeller;

$$\mu = \frac{\sqrt{V_i^2 + V_j^2}}{R\Omega} \quad (21)$$

Assuming that thrust is linearly proportional to the angular speed squared ($T = k\Omega^2$), where k is a scaling factor converting rotor angular speed to thrust, the total profile power is

$$P_P = \sum_{i=1}^{M}\left(\frac{Ncc_{d_{blade}}\rho R^4}{8}\Omega^3(1 + \mu^2)\right). \quad (22)$$

Let c_2 be $\frac{Ncc_{d_{blade}}\rho R^4}{8k^{3/2}}$ and c_3 be $\frac{Ncc_{d_{blade}}\rho R^2}{8k^{1/2}}$, then P_p can be written as

$$P_P = c_2 T^{3/2} + c_3\left(V_i^2 + V_j^2\right)T^{1/2}. \quad (23)$$

S5.3 Parasitic Power

The parasitic power (P_d) is the power required to overcome the drag force resulting from the movement of the quad-copter body through the air.

$$P_d = \sum F^b_{drag} V^b \tag{24}$$

where F^b_{drag} is the drag force on the quad-copter body, and V^b is the airspeed vector acting on the body frame.

$$F^b_{drag} = \frac{1}{2} C^b_d \rho A^b_{ref} (V^b)^2 \tag{25}$$

where C^b_d is the drag coefficient of the drone's body and A^b_{ref} is the reference area.
Therefore,

$$P_d = \sum \frac{1}{2} C^b_d \rho A^b_{ref} (V^b)^3 \tag{26}$$

and is the sum of the parasitic power of all surfaces, P_{di}, P_{dj} and P_{dk}.

S5.4 Ancillary Power

The ancillary power (P_a) is the power required to run all the electronic devices and sensors used for the flight navigation systems and data collection.

Although P_a may vary under extreme operation conditions, such as extremely high and/or low temperatures, it has been assumed that P_a will be relatively constant throughout the flight as long as the number and specifications of the electronic devices and sensors remain the same. Moreover, our data shows that the ancillary power varied between 4 W and 9 W, which corresponds to 1% to 2% of the average power of a flight with vertical movement (approximately 400 W).

S6. Quadratic Programming

Quadratic program (QP) was used to estimate coefficients c_1 to c_6 through the following form[2]

$$\min_x \frac{1}{2} x^T P x + q^T x \tag{27}$$

$$\text{subject to } \begin{bmatrix} Gx \leq h \\ Ax = b \end{bmatrix} \tag{28}$$

Let M be the matrix formed by our B values:

$$M = \begin{bmatrix} B_{11} & B_{21} & B_{31} & B_{41} & B_{51} & B_{61} & 1 \\ B_{12} & B_{22} & B_{32} & B_{42} & B_{52} & B_{62} & 1 \\ \vdots & \vdots & \vdots & \vdots & \vdots & \vdots & \vdots \\ B_{1n} & B_{2n} & B_{3n} & B_{4n} & B_{5n} & B_{6n} & 1 \end{bmatrix}$$

and x be the column matrix formed by our C values:

$$x = \begin{bmatrix} c_1 \\ c_2 \\ \vdots \\ c_7 \end{bmatrix}$$

The estimated power can be then calculated by multiplying Mx

$$P_{estimated} = M.x = \begin{bmatrix} B_{11} & B_{21} & B_{31} & B_{41} & B_{51} & B_{61} & 1 \\ B_{12} & B_{22} & B_{32} & B_{42} & B_{52} & B_{62} & 1 \\ \vdots & \vdots & \vdots & \vdots & \vdots & \vdots & \vdots \\ B_{1n} & B_{2n} & B_{3n} & B_{4n} & B_{5n} & B_{6n} & 1 \end{bmatrix} \begin{bmatrix} c_1 \\ c_2 \\ \vdots \\ c_7 \end{bmatrix}$$

[2] The approach used to solve the quadratic program followed the examples provided at https://scaron.info/blog/quadratic-programming-in-python.html

Let the measured power can be written as:

$$P_{measured} = \begin{bmatrix} P_1 \\ P_2 \\ \vdots \\ P_n \end{bmatrix}$$

The main objective function of the optimization problem is to minimize:

$$minimize \left|\left| \begin{bmatrix} B_{11} & B_{21} & B_{31} & B_{41} & B_{51} & B_{61} & 1 \\ B_{12} & B_{22} & B_{32} & B_{42} & B_{52} & B_{62} & 1 \\ \vdots & \vdots & \vdots & \vdots & \vdots & \vdots & \vdots \\ B_{1n} & B_{2n} & B_{3n} & B_{4n} & B_{5n} & B_{6n} & 1 \end{bmatrix} \begin{bmatrix} c_1 \\ c_2 \\ \vdots \\ c_6 \end{bmatrix} - \begin{bmatrix} P_1 \\ P_2 \\ \vdots \\ P_n \end{bmatrix} \right|\right|^2$$

The parameters in x must be positive, so it is necessary to add a constrain:

$$subject\ to \begin{bmatrix} -1 & 0 & 0 & 0 & 0 & 0 & 0 \\ 0 & -1 & 0 & 0 & 0 & 0 & 0 \\ 0 & 0 & -1 & 0 & 0 & 0 & 0 \\ 0 & 0 & 0 & -1 & 0 & 0 & 0 \\ 0 & 0 & 0 & 0 & -1 & 0 & 0 \\ 0 & 0 & 0 & 0 & 0 & -1 & 0 \\ 0 & 0 & 0 & 0 & 0 & 0 & -1 \end{bmatrix} \begin{bmatrix} c_1 \\ c_2 \\ c_3 \\ c_4 \\ c_5 \\ c_6 \\ c_7 \end{bmatrix} \leq \begin{bmatrix} 0 \\ 0 \\ 0 \\ 0 \\ 0 \\ 0 \\ 0 \end{bmatrix}$$

In other words, the optimization problem can be written as:

$$minimize\ ||Mx - b||^2$$

$$subject\ to : [Gx \leq h]$$

where G is a 7x7 identity matrix multiplied by -1, and h is a 7x1 zero matrix.
$||Mx - b||^2$, where b = $P_{measured}$, can be expanded as:

$$\begin{aligned} ||Mx - P||^2 &= (Mx - b)^T (Mx - b) \\ &= x^T M^T M x - x^T M^T b - b^T M x + b^T b \\ &\propto \left(\frac{1}{2}\right) x^T M^T M x - \left(\frac{1}{2}\right) x^T M^T b - \left(\frac{1}{2}\right) b^T M x \\ &= \left(\frac{1}{2}\right) x^T (M^T M) x + (-M^T b)^T x \end{aligned}$$

Which enables us to write the optimization problem in the quadratic standard, with $P = (M^T M)$, $q = (-M^T b)$, $G = -[I]_{7x7}$ and $h = [0]_{7x1}$

S7. Linear Regression

Here we describe the steps used to perform the linear regression and calculate the coefficients b_1, b_2, and R^2[5] used in the main method.

Let $x = \frac{m^{1.5}}{\sqrt{\rho}}$ and $y = \bar{P}$, where m is the total mass of the drone (including payload), ρ is the air density and $\bar{P}$ is the average power observed during a flight regime.

$$\bar{x} = \frac{\sum_{i=1}^{n} x_i}{n}$$

$$\bar{y} = \frac{\sum_{i=1}^{n} y_i}{n}$$

The sum of squares (S_{xx}) and cross-products (S_{xy}) are calculated as

$$S_{xx} = \sum_{i=1}^{n} (x_i - \bar{x})^2$$

$$S_{xy} = \sum_{i=1}^{n} (x_i - \bar{x})(y_i - \bar{y})$$

The linear coefficients b_1 and b_2 are then calculated as

$$b_1 = \frac{S_{xy}}{S_{xx}}$$

$$b_0 = \bar{y} - b_1 \bar{x}$$

The total sum of squares (SS_{tot}) and the regression sum of squares (SS_{reg}) are calculated as

$$SS_{tot} = \sum_{i=1}^{n} (y_i - \bar{y})^2$$

$$SS_{reg} = b_1^2 S_{xx}$$

Finally, the coefficient of determination (R^2) is obtained as

$$R^2 = \frac{SS_{ref}}{SS_{tot}}$$

S8. Machine Learning approach

While regression trees are simple and interpretable, they do not have good generalization properties: if the tree is too deep, it overfits to training data, and if it is too shallow, it does not capture complex structures. Boosting is an ensemble technique that sequentially fits weak regression trees, creating a powerful learner. Each new weak learner tries to correct the errors of the previous learners by targeting the residuals of prior models. `XGBoost` is a library that optimizes speed and performance of gradient boosted trees algorithms. In addition to the advantages of the gradient boosting algorithm, `XGBoost` also adds useful features that the user can control when running the model, such as selecting the learning rate, using a stochastic approach via subsampling, and adding regularization to improve generalization.

We use `XGBoost` as a gold standard when evaluating the energy model performance. Our modeling approach via `XGBoost` consists of the following steps:

1. Fix a regime and a set of hyperparameter values to be tuned. We varied the following hyperparameters: learning rate (0.01, 0.05, 0.1), maximum depth of each weak learner (3, 5, 8), minimum loss reduction required to make another partition (0, 1, 5).
   (a) Run `XGBoost` on the training data, using squared loss as the objective function. Our response variable and features are the same used in the first principles energy model. We used the stochastic approach by subsampling 80% of the data at each tree fitting. Our algorithm used up to 1000 trees; however, if there was no significant increase in performance after 50 trees, the algorithm stopped. Test error estimation for hyperparameter tuning was performed via 5-fold cross validation.
   (b) Plot number of trees (up to 1000) versus training error and estimated test error via 5-fold cross validation.
2. Select hyperparameter combination to be used for each regime (number of trees, learning rate, maximum depth, minimum loss reduction) by choosing a low test error, low generalization error[3], and low standard errors. The selected hyperparameter values are in Table S1.
3. Fit selected model for each regime using training data and evaluate its performance by computing errors (ARE) using test data.

	Take Off	Cruise	Landing
learning rate	0.05	0.1	0.1
maximum depth	3	3	3
minimum loss reduction	0	5	5
number of trees	250	200	250

Table S1: Selected hyperparameter values for each flight regime.

[3]Generalization error is the difference between training error and test error.

S9. Prediction power of the energy model

Figure S12 presents the Absolute Relative Error on the test set for the three models. Model 1 is described in the main paper. Model 2 corresponds to a model including the four forms of power described in equation 5 of the section "First Principles Analysis" of this Supplementary Information. Between the two proposed models, Method 1 showed considerably lower average relative error of 2.1% when compared to Method 2 (with average relative error of 10%). Given its high flexibility and working as our optimal reference for predictive power, `XGBoost` attained the lowest average relative error (2%) as expected. Method 1's absolute relative error is fairly close to `XGBoost`, supporting Method 1 as a good model both for predictive power and interpretability.

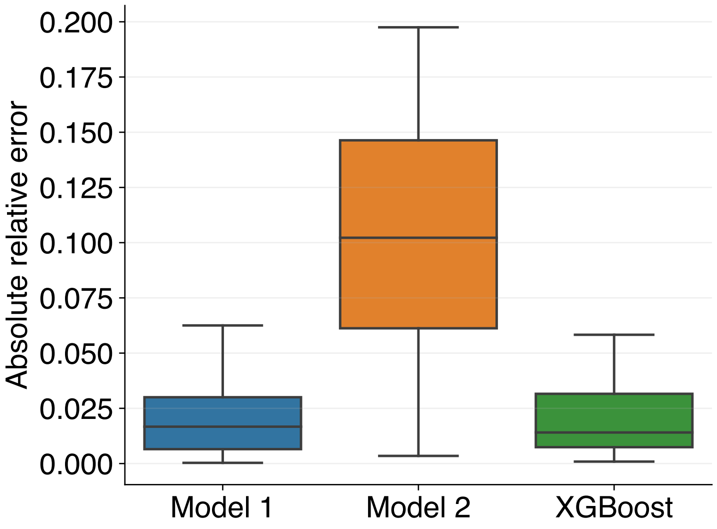

Figure S12: Boxplots of Absolute Relative Error (ARE) computed based on the test set of flights using Method 1, Method 2, and `XGBoost`.

S10. Parameters used in the sensitivity analysis

Table S2 provides the delivery intensity values assumed for lower, base and upper cases.

Table S2: Values assumed for delivery intensity for lower, base, and upper cases.

**Vehicle Class**	**Delievery intensity [packages/km] (lower, base, upper)**
Medium duty truck	(1.5, 2.1, 5)
Small diesel van	(1.5, 3.5, 5)
Medium duty electric truck	(1.5, 2.1, 5)
Small electric van	(1.5, 3.5, 5)
Electric cargo bicycle	(0.25, 1, 3)
Quad-copter drone	(0.125, 0.25, 0.5)

Table S3 and Table S4 provide the GHG emissions per km and per package, respectively, of the electric vehicles assessed in this study.

Table S3: Life cycle GHG emissions by vehicle per km for each EPA subregion [gCO_2e/km]. Direct emissions from EPA Subregions,[6] upstream electricity and battery emissions from[7]

**Subregion**	**Medium duty electric truck**	**Small electric van**	**Electric cargo bicycle**	**Quad-copter drone**
AKGD	742.5	326.1	22.4	16.7
AKMS	838.1	367.6	24.9	18.7
AZNM	767.0	336.7	23.0	17.2
CAMX	592.2	260.8	18.4	13.5
ERCT	732.4	321.7	22.1	16.5
FRCC	594.4	261.7	18.5	13.5
HIMS	852.0	373.7	25.3	19.0
HIOA	954.6	418.3	28.0	21.2
MROE	893.5	391.7	26.4	19.9
MROW	981.7	430.0	28.7	21.8
NEWE	533.3	235.2	16.8	12.2
NWPP	905.6	397.0	26.7	20.2
NYCW	573.8	252.8	17.9	13.1
NYLI	713.4	313.4	21.6	16.1
NYUP	530.2	233.8	16.8	12.1
PRMS	912.8	400.1	26.9	20.3
RFCE	702.1	308.5	21.3	15.8
RFCM	940.5	412.1	27.6	20.9
RFCW	981.5	430.0	28.7	21.8
RMPA	904.2	396.3	26.7	20.1
SPNO	1059.1	463.6	30.8	23.5
SPSO	837.1	367.2	24.9	18.7
SRMV	655.1	288.1	20.1	14.8
SRMW	1009.1	441.9	29.5	22.4
SRSO	752.0	330.2	22.6	16.9
SRTV	837.2	367.2	24.9	18.7
SRVC	746.3	327.7	22.5	16.8
US	782.5	343.5	23.4	17.5

Table S4: Life cycle GHG emissions by vehicle per package for each EPA subregion [$gCO_2e/package$]. Direct emissions from EPA Subregions,[6] upstream electricity and battery emissions from,[7] and base-case delivery intensity as provided in S2

Subregion	Medium duty electric truck	Small electric van	Electric cargo bicycle	Quad-copter drone
AKGD	353.6	93.7	22.4	66.7
AKMS	399.1	105.6	24.9	74.9
AZNM	365.3	96.8	23.0	68.8
CAMX	282.0	74.9	18.4	53.8
ERCT	348.7	92.4	22.1	65.8
FRCC	283.0	75.2	18.5	54.0
HIMS	405.7	107.4	25.3	76.1
HIOA	454.6	120.2	28.0	84.9
MROE	425.5	112.6	26.4	79.6
MROW	467.5	123.6	28.7	87.2
NEWE	253.9	67.6	16.8	48.8
NWPP	431.3	114.1	26.7	80.7
NYCW	273.2	72.6	17.9	52.3
NYLI	339.7	90.1	21.6	64.2
NYUP	252.5	67.2	16.8	48.5
PRMS	434.7	115.0	26.9	81.3
RFCE	334.3	88.7	21.3	63.2
RFCM	447.9	118.4	27.6	83.7
RFCW	467.4	123.6	28.7	87.2
RMPA	430.6	113.9	26.7	80.6
SPNO	504.3	133.2	30.8	93.8
SPSO	398.6	105.5	24.9	74.8
SRMV	311.9	82.8	20.1	59.2
SRMW	480.5	127.0	29.5	89.5
SRSO	358.1	94.9	22.6	67.5
SRTV	398.7	105.5	24.9	74.8
SRVC	355.4	94.2	22.5	67.0
US	372.6	98.7	23.4	70.1

Supplemental References

[1] Rotaru, C. and Todorov, M. (2017). Helicopter Flight Physics. In Flight Physics - Models, Techniques and Technologies. InTech. 10.5772/intechopen.71516.

[2] Beard, R. W. and McLain, T. W. (2012). Small unmanned aircraft: Theory and practice. ISBN 9780691149219.

[3] DJI. MATRICE 100 SPECS. Available at `https://www.dji.com/matrice100/info`.

[4] Liu, Z., Sengupta, R., and Kurzhanskiy, A. (2017). A power consumption model for multi-rotor small unmanned aircraft systems. In 2017 International Conference on Unmanned Aircraft Systems (ICUAS), pages 310–315. IEEE. ISBN 978-1-5090-4495-5. 10.1109/ICUAS.2017.7991310.

[5] Baron, M. (2013). Probability and Statistics for Computer Scientists. In Probability and Statistics for Computer Scientists, chapter 11. Chapman and Hall/CRC, second edition edition. ISBN 9780429108761. 10.1201/B14800.

[6] US EPA (2020). Emissions & Generation Resource Integrated Database (eGRID).

[7] Laboratory, A. N. (2020). GREET.
