# Supplementary material for: Drone flight data reveal energy and greenhouse gas emissions savings for very small package delivery
Source: Patterns (N Y). 2022 Aug 5;3(8):100569. doi: 10.1016/j.patter.2022.100569 (PMC9403403; doi:10.1016/j.patter.2022.100569)
Supplement: Document S2. Article plus supplemental information [file mmc2.pdf]

Article

Patterns

Drone flight data reveal energy and greenhouse gas emissions savings for very small package delivery

Graphical abstract

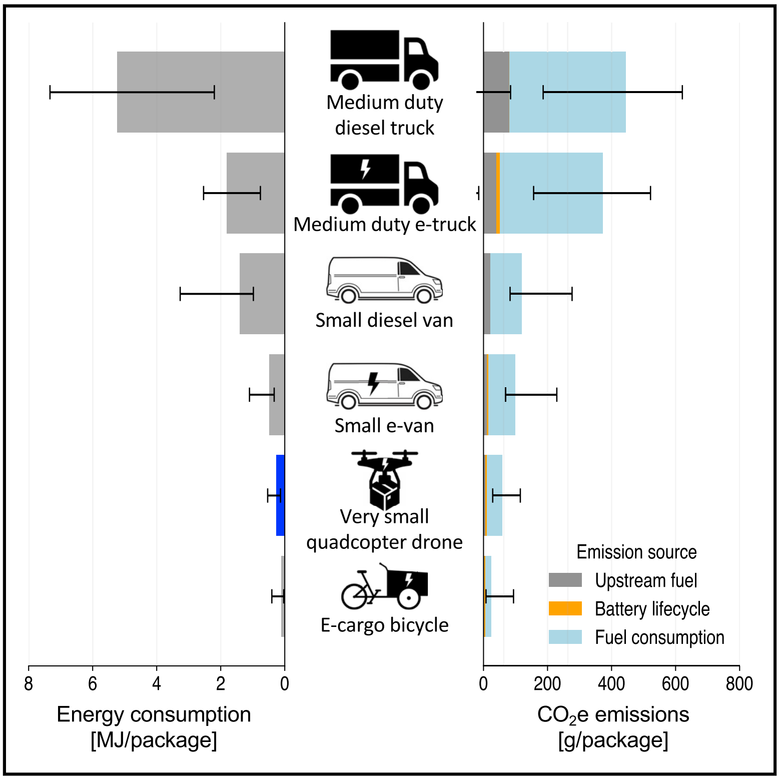

Highlights

- Drones can have up to 94% lower energy consumption per package than other vehicles
- Small quadcopter drones consume approximately 0.08 MJ/km and emit 70 gCO_2e/package
- Total energy consumption can be reduced by 34% by reducing vertical takeoff
- GHG emissions are up to 93% higher in regions with a more carbon-intensive grid mix

Authors

Thiago A. Rodrigues, Jay Patrikar, Natalia L. Oliveira, H. Scott Matthews, Sebastian Scherer, Constantine Samaras

Correspondence

tarodrig@andrew.cmu.edu (T.A.R.), csamaras@cmu.edu (C.S.)

In brief

We help stakeholders and researchers understand the energy use of package-delivery drones and provide an open model based on 188 delivery-drone flights with a range of payloads, speeds, and altitudes. We show the impact of the cruise speed and payload mass on the drone's range, provide generalizable coefficients for others to estimate drone energy use, and show that energy per package delivered by drones can be up to 94% lower than conventional transportation modes.

Rodrigues et al., 2022, Patterns *3*, 100569
August 12, 2022 © 2022 The Author(s).
https://doi.org/10.1016/j.patter.2022.100569

CellPress

Patterns

CellPress
OPEN ACCESS

Article

Drone flight data reveal energy and greenhouse gas emissions savings for very small package delivery

Thiago A. Rodrigues,[1,*] Jay Patrikar,[2] Natalia L. Oliveira,[3,4] H. Scott Matthews,[1] Sebastian Scherer,[2] and Constantine Samaras[1,5,*]
[1]Department of Civil and Environmental Engineering, Carnegie Mellon University, 5000 Forbes Avenue, Pittsburgh, PA 15213, USA
[2]Robotics Institute, Carnegie Mellon University, 5000 Forbes Avenue, Pittsburgh, PA 15213, USA
[3]Department of Statistics and Data Science, Carnegie Mellon University, 5000 Forbes Avenue, Pittsburgh, PA 15213, USA
[4]Machine Learning Department, Carnegie Mellon University, 5000 Forbes Avenue, Pittsburgh, PA 15213, USA
[5]Lead contact
*Correspondence: tarodrig@andrew.cmu.edu (T.A.R.), csamaras@cmu.edu (C.S.)
https://doi.org/10.1016/j.patter.2022.100569

THE BIGGER PICTURE The growing demand for fast, contactless deliveries has been driving firms to experiment with automated package-delivery vehicles that can avoid urban traffic and rapidly reach customers. Widespread adoption of uncrewed aerial vehicles (UAVs) to replace a portion of first/last-mile truck pickups and deliveries could reshape transportation energy by changing demand patterns and shifting fuel use from fossil fuels to electricity. There is a need to optimize drones and systems to maximize energy productivity. Here, we help stakeholders and researchers understand the energy use of package-delivery drones and provide an open model based on 188 delivery-drone flights with a range of payloads, speeds, and altitudes. We show the impact of the cruise speed and payload mass on the drone's range, provide generalizable coefficients for others to estimate drone energy use, and show that the energy per package delivered by drones can be up to 94% lower than conventional transportation modes.

1 2 3 4 5 **Proof-of-Concept:** Data science output has been formulated, implemented, and tested for one domain/problem

SUMMARY

Uncrewed aerial vehicles (UAVs) for last-mile deliveries will affect the energy productivity of delivery and require new methods to understand energy consumption and greenhouse gas (GHG) emissions. We combine empirical testing of 188 quadcopter flights across a range of speeds with a first-principles analysis to develop a usable energy model and a machine-learning algorithm to assess energy across takeoff, cruise, and landing. Our model shows that an electric quadcopter drone with a very small package (0.5 kg) would consume approximately 0.08 MJ/km and result in 70 g of CO_2e per package in the United States. We compare drone delivery with other vehicles and show that energy per package delivered by drones (0.33 MJ/package) can be up to 94% lower than conventional transportation modes, with only electric cargo bicycles providing lower GHGs/package. Our open model and coefficients can assist stakeholders in understanding and improving the sustainability of small package delivery.

INTRODUCTION

Achieving large improvements in the energy productivity of freight transportation is challenging, especially in the overwhelmingly petroleum-powered transport sector where medium and heavy trucks in the United States comprise 24% of transportation energy use. This sector is responsible for 37% of transportation-related greenhouse gas (GHG) emissions, while light-duty vehicles comprise 57% of transportation GHG emissions and 64% of transportation energy use. In addition, transportation remains a large source of nitrogen oxides (NOxs) and other air pollutants.[1] However, the way that consumers are obtaining goods in the United States is changing rapidly.[2]

This is an open access article under the CC BY license (http://creativecommons.org/licenses/by/4.0/).

Even before COVID-19, the growing demand[3] for fast, contactless deliveries has been driving firms to experiment with automated package-delivery vehicles, such as uncrewed aerial vehicles (UAVs), that can avoid traffic in urban centers.[4,5] Initial survey data of 483 customers in Portland, Oregon by Pani et al.[6] show that COVID-19 is contributing to an environment where more than 60% of online customers are willing to pay extra to receive their packages using autonomous delivery robots. Nevertheless, along with technology and policy challenges, increased shipping costs is a limitation for the adoption of autonomous delivery vehicles.[7]

The appeal of delivery robots also reflects new physical distancing demands to avoid the spread of coronavirus in product deliveries,[8] and as autonomous delivery technologies advance, new companies emerge to compete for this market niche.[9] At the same time, alternative transport modes, such as electric cargo bicycles, are becoming cost-effective alternatives to delivery trucks for short-distance deliveries,[10] drastically reducing the CO_2 emissions of last-mile delivery in dense metropolitan areas.[11] With the increased electrification of delivery vehicles, the energy consumption and environmental impacts of the transportation sector are expected to change drastically over the coming years,[12,13] and technology, policy, and demand are primary drivers. Widespread adoption of UAVs to replace a portion of first/last-mile truck pickups and deliveries could reshape this sector by changing demand patterns and shifting fuel demands from fossil fuels to electricity. Autonomous delivery robots are coming to the transportation sector, but how these vehicles and systems could be designed to maximize energy productivity is less clear.

So far, a few studies have estimated the energy consumption of quadcopter vehicles, and the energy estimations vary considerably among the different methods used.[14] Some studies have created models based on theoretical principles,[15–23] while others have developed models based on regression models built on small flight samples.[17,21,24] Finally, a comparison of the energy consumption and GHG emissions between package-delivery UAVs and different transportation modes have been estimated by a few studies,[20,25–27] but alternative emerging delivery modes, such as electric cargo bicycles, are not included. Our study builds a model based on a much larger empirical test sample of a drone operating at altitudes between 25 and 100 m and with drone ground speeds that vary from 4 to 12 m/s in flights operating under wind conditions varying from 2 to 16 knots. We show that, within these limits, the ground speed and the wind condition impact little on the average energy consumption of the quadcopter drone and that the induced power at a hover and no-wind condition can be used as a good estimator of the average power experienced by the aircraft throughout the flight.

The adoption of a multirotor UAV was motivated by the commercial use of similar aircraft by last-mile delivery companies. For example, the company SpeedBird Aero has used a multirotor with a capacity of 2 kg to deliver food in Latin America.[28] The Irish drone startup Manna[29] uses a quadcopter drone to carry payloads varying between 2 and 4 kg.[30] The DJI Matrice 100 (M100) used in this study is a smaller quadcopter drone and was tested with a maximum payload of 0.5 kg. The M100 carrying a very small payload is likely smaller than purpose-built package-delivery drones for larger packages, which require future work to understand specific energy characteristics. However, we believe that the results using the M100 can provide important information to researchers and industry professionals working with UAVs for last-mile deliveries for very small packages such as medical deliveries, critical parts, or other time-sensitive payloads. Here, we help stakeholders and researchers understand the energy use of uncrewed aerial package-delivery drones. We provide an energy model based on extensive empirical data from 188 flights of a quadcopter drone M100, from which we developed a high-resolution dataset of package-delivery-drone energy use.[31] In addition, we develop an algorithm that automatically identifies the flight regime across takeoff, cruise, and landing. We show the impact of the cruise speed and payload mass on the drone's range and provide energy-use coefficients. We use our energy model to compare the energy consumption and GHG emissions of the drone with delivery trucks, delivery vans, and electric cargo bicycles on a distance and package basis. We show how the drone's emissions differ regionally in the US according to the electricity mix. We perform a sensitivity analysis on the energy consumption on a distance basis (MJ/km) and show the minimum drone energy consumption required to match different vehicles, which can help inform drone designers for future efficient UAVs. Finally, we show the delivery intensity required to match the GHG emissions of the drone for each region of the US.

RESULTS

We conducted a first-principles analysis and developed a model to estimate the energy required to power a quadcopter. Each of the flight regimes (takeoff, cruise, and landing) were modeled separately, so each energy model was treated as a model class and three different optimal models from that class were selected, one per regime. In order to fairly compare the model classes' performance and avoid overfitting, we split the data into train and test folds following a stratification strategy by flight ID number. With 120 flights, the training fold was used to estimate the parameters of each model, which were then applied to the remaining 68 flights from the test fold in order to evaluate the performance of the energy models on unseen data.

Energy model derived from flights

Our energy (E) model uses the induced power (P_i), which is the power required to overcome gravity in a hover-no-wind situation, as a parameter estimator of the average power observed throughout the flight.

$$E = \left(b_1' P_i + b_0\right) t, \quad \text{(Equation 1)}$$

where t is the flight duration, and b_1' and b_0 are coefficients that linearly correlate P_i and the average power throughout the flight.

The P_i, used in Equation 1, is calculated as

$$P_i = \frac{(mg)^{1.5}}{\sqrt{2\rho A}}, \quad \text{(Equation 2)}$$

where m is the total mass of the drone (including the payload), g is the acceleration of gravity, ρ is the air density that we obtained

Table 1. Model coefficient ± bootstrap standard error

Coef.	Takeoff	Cruise	Landing
b_1	80.4 ± 2.6	68.9 ± 2.0	71.5 ± 1.7
b_0	13.8 ± 18.9	16.8 ± 15.0	−24.3 ± 12.5
R^2	0.84	0.85	0.90

from the closest airport station (KAGC), and A is the total area under the propellers. Our experiment focused on one type of quadcopter drone; hence, A is constant here.

Combining Equation 2 and Equation 1,

$$E = \left(b_1\frac{m^{1.5}}{\sqrt{\rho}} + b_0\right)t, \quad \text{(Equation 3)}$$

where b_1 includes the constants A and g. The estimated coefficients and their standard errors are shown in Table 1.

The coefficients shown in Table 1 were obtained by performing a linear regression between P_i and the average power observed throughout each of the 120 flights. The results were then applied to the remaining flights, and the absolute relative error was 2.1% on average, proving the accuracy of the energy model in terms of estimation of energy consumption.

With the energy model validated, we estimated the energy consumption of a package delivered by a small quadcopter drone. Our energy model suggests that within the speed range tested, the average power consumption of a quadcopter does not vary considerably with the speed during cruise, which has also been observed with our dataset (Figure 1). The speed does affect total flight time, and hence for the drone and range of speeds we tested, total power consumption of each delivery will be higher when flight speeds are lower.

Drone energy use and GHG emissions

The GHG emissions of a package delivery by drone will depend on the total electricity needed for the delivery and the emissions intensity of the regional electricity grid.[20] Our analysis shows that variations in the cruise speed have a large impact on the total energy consumption per trip and, consequently, the range of the drone. Because the total time of flight is reduced as the speed increases, a faster speed (with normal operating parameters) for a quadcopter generally enables longer delivery distances for the same amount of energy. We show the influence of speed, payload, and delivery distance on total energy consumption in Figure 2A.

We then calculated the GHG emissions per package delivered based on the US regional non-baseload electricity GHG emissions from the US Environmental Protection Agency,[32] upstream electricity generation emissions, and battery life cycle emissions. In Figure 2B, we show the GHG emissions per package delivery as a function of the delivery distance. We show the results for the fastest speed evaluated, 12 m/s; however, the model enables evaluation across the full range of speeds, altitudes, and payloads.

We illustrate the impacts of the GHG intensity of a regional electricity grid on drone package delivery GHGs in Figure 3. Using regional non-baseload emissions factors, we show that a drone package delivery in the carbon-intensive central Midwest would emit up to 93% more CO_2e per km traveled (23.5 g CO_2e/km) compared with regions with cleaner grid mixes such as New York, which would result in drone emissions of 12.1 g CO_2e/km.

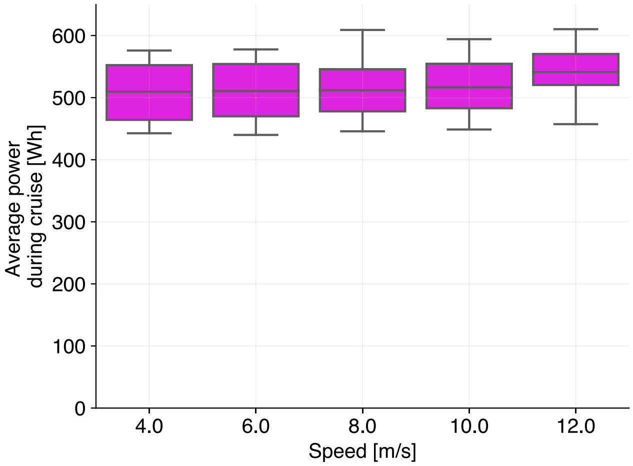

Figure 1. Our data show that for the range of cruise speeds tested, there is no practical variation in the drone's average power consumption (approximately 5% variation between the median of flights at 4 and 12 m/s)

Comparison between different transportation modes

We compared the energy consumption of quadcopter drones against diesel and electric medium-duty trucks and small vans and electric cargo bicycles.

The total energy consumption per distance of small quadcopter drones is among the lowest across transportation modes, as the vehicle is small, light, and has lower payload capacity (Figure 4A). Figure 4B shows the energy consumption per package of drone-equivalent deliveries, i.e., assuming that all packages delivered by the other modes are within the payload and space capacity of a small quadcopter drone. On an energy consumption per package basis, small quadcopter drones and electric cargo bicycles are among the most energy-efficient modes for small package delivery. The number of stops per kilometer and the number of packages delivered per stop varies according to the transportation mode and delivery density (highly dense areas are more likely to have more stops and packages delivered per kilometer).

Similarly, an analysis of the GHG emissions of the fuel of each transportation mode shows that quadcopter drones and electric cargo bicycles are among the most efficient vehicles in g of CO_2e per km (Figure 5A) and a competitive alternative in terms of GHG emissions per package (Figure 5B). On the other hand, it is important to note that small drones are considerably limited in terms of weight and volume of the packages transported. Therefore, an analysis of the energy consumption and GHG emissions on a per metric ton-km basis in Figure S1 shows that small drones are the most energy-intensive vehicles. Also, local airspace regulations, such as not flying over people and/or motor vehicles,[33] could impose longer delivery routes, were not considered in this study, and could potentially increase the

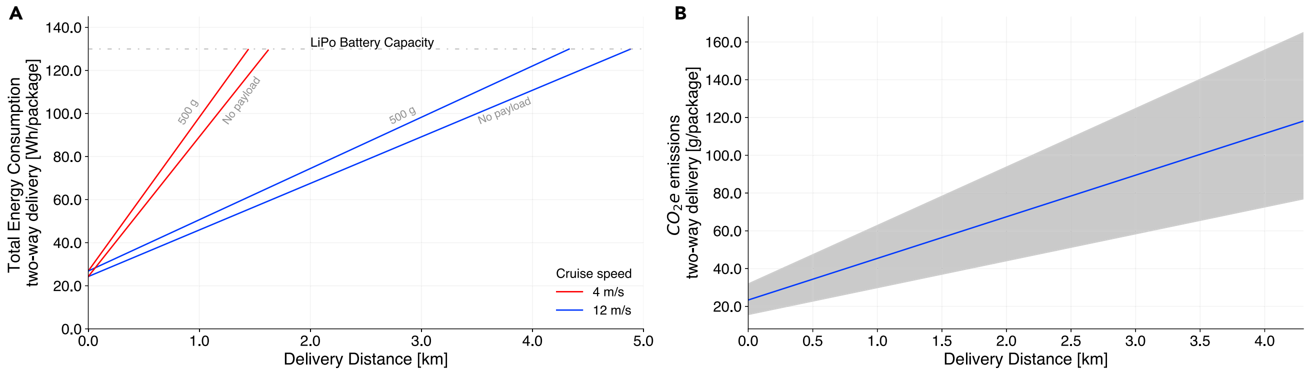

Figure 2. Small drone total energy consumption and greenhouse gas emissions by distance

(A) Total energy consumption by distance of delivery varying payload mass and cruise speed. Total trip energy is higher at lower speeds for a fixed distance.

(B) CO_2e emissions of delivering a payload of 0.5 kg at a cruise speed of 12 m/s, the fastest speed evaluated, based on the delivery distance. The uncertainty area corresponds to US regional grid emissions factors. The total energy and CO_2e correspond to takeoff, cruise from the origin to destination, and landing loaded and takeoff, cruise from destination to origin, and landing empty. As an energy limitation, the nominal capacity of an LiPO TB48D battery is 130 Wh. Results shown here have an altitude during cruise of 100 m, takeoff speed of 2.5 m/s, and landing speed of 2 m/s.

drone's energy consumption and GHG emissions per package delivered.[34]

We compared our results with values provided by the United Parcel Service (UPS). In 2019, UPS reported the energy intensity for US Domestic Package operations was 28 MJ/package, from which ground vehicles represented approximately 9.5 MJ/package or 34% (airline fuel, facility heating fuel, and indirect energy correspond to 60%, 3%, and 3%, respectively), with GHG emissions (CO_2e) intensity of 1 kg/package.[35] It is important to note that these values encompass the entire ground fleet rather than only last-mile delivery.

Finally, assuming a base case where the drone delivers an average of 0.25 packages per km (one-way delivery distance of 2 km) and consumes 0.08 MJ/km, we calculated the minimum number of packages per km required by each vehicle to match the drone's energy consumption and CO_2e per package. Table 2 shows that a medium-duty diesel truck would require approximately 34 packages per km to meet the drone's performance, which would correspond to having 200 packages delivered in a route of less than 6 km. On the other hand, a small electric van would require a delivery intensity of approximately five packages per km, or a 39-km route to deliver 200 packages, which could potentially be achieved in dense urban centers.

Moreover, Table 2 also shows the minimum energy consumption required for the drone to match each vehicle's energy consumption per package. To have a similar energy consumption per package delivered by a medium-duty diesel truck, a small quadcopter drone would need to consume 1.31 MJ/km (approximately 19 times more than our base-case estimate). Figure S2 provides a similar analysis considering that the GHG emissions showing that a diesel truck would need to deliver between 10 and 19 packages per km depending on the region of operation. Future work could estimate of the density of deliveries that would support operations with similar delivery density on a per-area

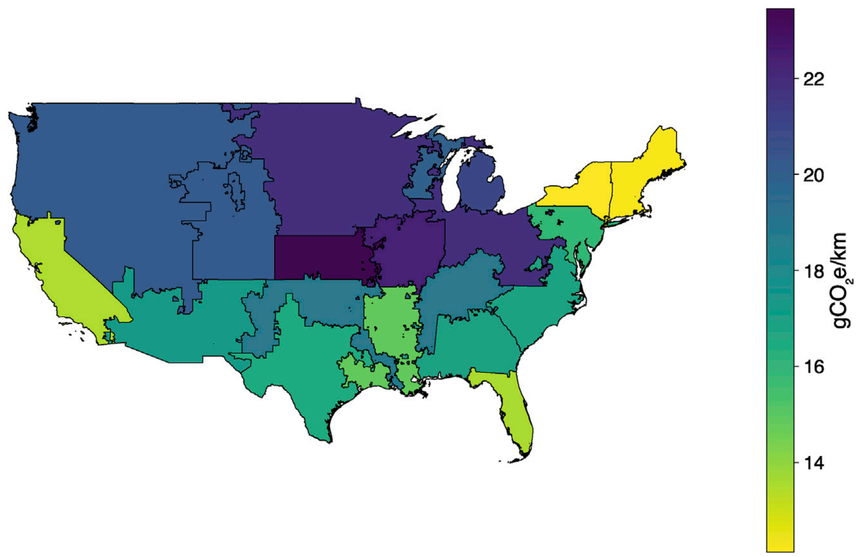

Figure 3. GHG emissions per km from a drone package delivery with a 0.5-kg payload and 2-km one-way delivery distance (4-km round trip), according to the sub-region's non-baseload electricity grid carbon intensity

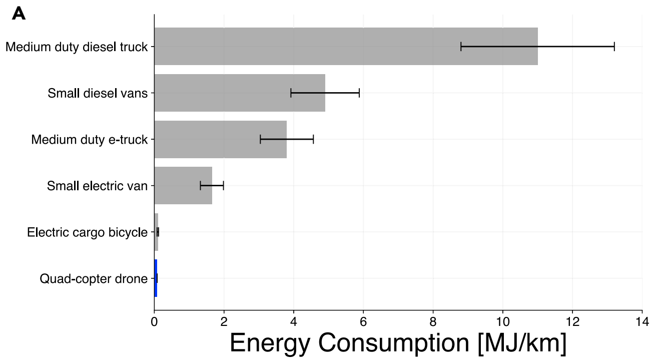

Figure 4. Energy consumption per distance and per package delivered for different transportation modes
Error bars represent variations in (A) driving styles and vehicle characteristics and (B) number of packages delivered per distance.

basis to understand which communities are likely to have energy and GHG savings with drone deliveries.

DISCUSSION

A small quadcopter drone, with a payload of 0.5 kg operating at a cruise speed of 12 m/s and a cruise altitude of 100 m, consumes approximately 0.08 MJ/km and generates 70.1 g of CO_2e per package when charged on average non-baseload US electricity, with a range of 48.5 g per package in New York (the cleanest US electricity region) to 93.8 g per package in the most carbon-intensive region in the central Midwest. Only electric cargo bicycles had a lower carbon footprint per package, from 16.8 g per package in New York, 23.4 g per package for the average US, and 30.8 g per package in the central Midwest. As the electricity grid gets cleaner over time, the carbon intensity of delivery with electricity-powered vehicles, whether drone, cargo bicycle, van, or truck, will continue to improve.

Our energy model has simple and accurate coefficients that can provide stakeholders and researchers with a drone-energy-consumption estimation for speeds below 12 m/s for similar small drones. However, at greater speeds or using drones with more surface area or mass, a more comprehensive energy-profile method could provide more accurate predictions.

The energy consumption of a very small, commercially available quadcopter drone with payload of 0.5 kg is comparable to the most energy-efficient modes of last-mile delivery when the total mass of delivery is not the main feature considered. For example, in delivery situations where small and light items with high added value, such as medical deliveries, critical packages, and small electronics, very small drones might become a competitive tool to reduce transportation emissions in large urban centers.[36] In these scenarios, we found that drones can reduce the energy consumption by 94% and 31% and GHG emissions by 84% and 29% per package delivered by replacing diesel trucks and electric vans, respectively. We also found that electric cargo bicycles had similar or lower GHGs per package than drones. We also found that the delivery intensity, i.e., the number of packages delivered per km, and the fuel carbon intensity are the main factors contributing to the drone's comparative energy and environmental performance.

Adding empirical tests to assess the impacts of varying the area of the propeller, the drone geometry and delivery intensity that were not included in this study could provide an important contribution to this field in future work. It is also important to note that the drone used to collect the data was not optimized to minimize energy consumption, which could further improve its efficiency. The drone used was designed for a very small package, and future studies should include heavier payloads.

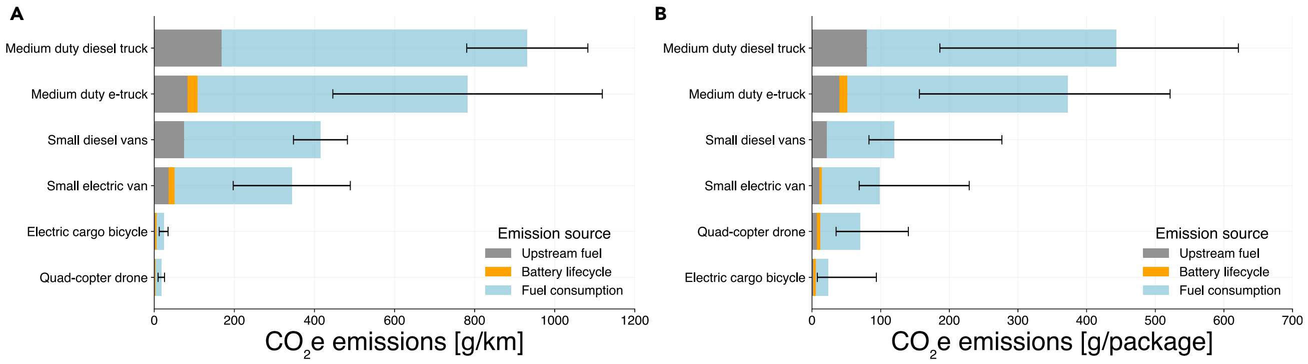

Figure 5. GHG emissions per distance and per package delivered for different transportation modes
Error bars (A and B) represent uncertainties due to variations on fuel carbon intensity, battery life cycle emissions, and number of packages delivered per distance.

Table 2. Delivery density of each mode to match the drone's energy consumption per package, and the minimum drone energy consumption to match the vehicle's energy consumption per package

Vehicle	Delivery density required to match drone energy consumption (package/km) (multiplier from base case)	Minimum energy consumption required for the drone (MJ/km)
Medium-duty diesel truck	33.8 (16.1×)	1.31
Small diesel van	15.0 (4.3×)	0.35
Medium-duty electric truck	11.7 (5.6×)	0.45
Small electric van	5.1 (1.5×)	0.12
Electric cargo bicycle	0.3 (0.3×)	0.03
Small quadcopter drone (base case)	0.25	0.07

Given the potential for improved energy productivity of delivery and reduced GHGs per package, very small package delivery by drone and electric cargo bicycles can play an important role in reducing the energy and climate impacts of package delivery.

EXPERIMENTAL PROCEDURES

Resource availability

Lead contact

Further information should be directed to and will be fulfilled by the lead contact, Dr. Samaras (csamaras@cmu.edu).

Materials availability

This study did not generate new unique reagents.

Data and code availability

All drone data are available at Figshare: https://doi.org/10.1184/R1/12683453, and all modeling codes are available at Zenodo: https://doi.org/10.5281/zenodo.6726991.

We collected data on 188 flights to assess the power profile of a package-delivery drone given a set of operational parameters (payload, altitude, and speed during cruise). The data, available at Rodrigues et al.,[37] and a data descriptor[31] provide the details of the experiment. In addition, we have developed an algorithm that separates the data into three different flight regimes: takeoff, cruise, and landing, in order to better understand the energy consumption profile during flight. We then perform a first-principles analysis to develop an energy model to estimate the drone's energy consumption. Finally, we use the energy model to compare the drone with different transportation models from an energy consumption and GHG emissions basis.

Empirical data collection from drone flight

We performed a series of flights to empirically measure the energy consumption of a quadcopter UAV. An experimental protocol was created and followed to ensure a reliable approach for data acquisition.[31]

An M100 quadcopter was equipped with an anemometer, current and voltage monitor, GPS, and accelerometer collecting data on wind speed and direction, battery current and voltage demand, and position, orientation, velocity, and acceleration. The flights were performed in a pre-established route with varying altitudes (25, 50, 75, and 100 m), speeds (4, 6, 8, 10, and 12 m/s), and payload mass (no payload, 250 g, and 500 g). The sensors and computer box attached to the drone weighed approximately 1,200 g. Each combination was repeated at least three times, totaling 188 flights. The data provided by each sensor were synchronized to a frequency of approximately 5 Hz using the ApproximateTime[38] message filter policy of Robot Operating System (ROS).

For a better understanding of the energy-consumption profile of each flight, we created an algorithm to automatically divide the data into three different flight regimes: takeoff, cruise, and landing (Figures S3–S6). The data available in Rodrigues et al.[37] were processed using this algorithm before the analysis described in the next sections.

First-principles analysis

The energy required to power a UAV can be estimated using a first-principles analysis based on helicopter aerodynamics.[39] First, we defined the working coordinate frames for a quadcopter drone (Figures S7–S9). Then, we assessed the power required to maintain the drone at a steady hover condition. Finally, we expanded the power analysis to include other power demands.

The main power demand of a drone is in the form of P_i. The P_i represents the power required to overcome the force of gravity in order to keep the aircraft in the air, and it can vary according to the flight maneuver.[39] The most basic way to estimate P_i is considering a hover condition without wind (Figure 6).

In that case, the thrust (T) equals the only force acting on the drone: its weight ($W = mg$),[39] and P_i can be estimated as

$$P_i = Tv_i, \qquad \text{(Equation 4)}$$

where v_i is the induced velocity.

During hover, v_i can be simplified as

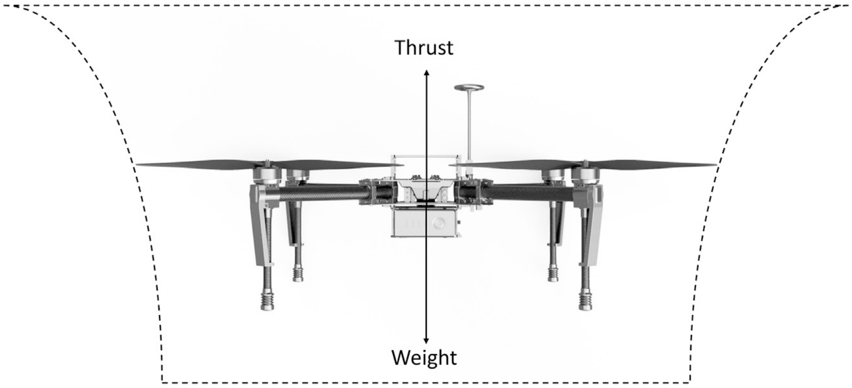

Figure 6. Drone flying at a hover and no-wind condition

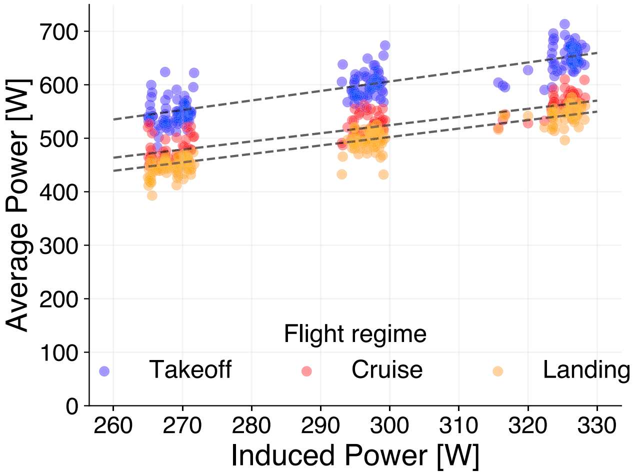

Figure 7. Relationship of induced power to drone average power of flights separated by flight regime

Payloads of 0 (left), 250 (center), and 500 g (right). R^2 are 0.84, 0.85, and 0.90 for takeoff, cruise, and landing, respectively.

$$v_i = \sqrt{\frac{T}{2\rho A}}, \quad \text{(Equation 5)}$$

where ρ is the air density and A is the total area covered by all four propellers.

Combining Equations 5 and 4

$$P_i = \frac{(T)^{3/2}}{\sqrt{2\rho A}} = \frac{(mg)^{1.5}}{\sqrt{2\rho A}}, \quad \text{(Equation 6)}$$

where m is the total mass of the drone and g is the gravitational acceleration.

More details of the first-principles analysis and an expanded first-principles energy model are available in the supplemental information (Figures S10 and S11).

Regression-based energy model

Our energy model inquires how effectively P_i can be used as an estimator for the energy consumed during a package-delivery flight. In such a case, the average power ($\overline{P}$) throughout the flight is modeled as a linear regression of the P_i

$$\overline{P} = b_1' P_i + b_0, \quad \text{(Equation 7)}$$

where b_1 and b_0 are the slope and intercept of the linear regression, respectively.

We can expand Equation 7 to account for the sum of the three flight regimes (Figure 7) and combine the area of the propeller (A) and gravity acceleration (g) from Equation 2 with coefficient b_1. Thus, the total energy consumption (E) is estimated as

$$E = \sum_{r \in \mathcal{R}, l \in \mathcal{L}} \left(b_1^{(r,l)} \frac{m^{1.5^{(l)}}}{\sqrt{\rho}} + b_0^{(r,l)} \right) t^{(r,l)} \quad \text{(Equation 8)}$$

for $\mathcal{R} = \{takeoff, cruise, landing\}$ and $\mathcal{L} = \{loaded, unloaded\}$.

The step-by-step calculation used to compute b_0, b_1, and R^2 can be found in the supplemental information under the section linear regression.

Machine learning for comparison

Evaluating whether the model's performance is good given the available measurements cannot be inferred from its performance alone. Therefore, we compare the predictive power of the energy model with a flexible nonlinear algorithm,[40] `XGBoost`, available in the programming environment `R`. This boosted tree algorithm prioritizes predictive power against interpretability, and it is appropriate for predictive performance given the available features. If our energy model presents similar accuracy to `XGBoost`, it indicates that the parametric and functional restrictions we have made for the energy model development are suitable.

We fitted a gradient boosted tree algorithm, XGBoost.[40] The algorithm was separately trained for each flight regime with a quadratic loss function, and for all regimes, we used 80% as a subsample ratio of both features and observations for each tree. For hyperparameter tuning, we varied learning rate, maximum tree depth, and regularization parameter γ in a grid search approach. 5-fold cross-validation (CV) was used for error estimation; for tuning only, we compared the absolute relative error (ARE; Equation 9) instead of the quadratic error. We fixed a regime and a set of hyperparameter values to be tuned then varied the following hyperparameters: learning rate (0.01, 0.05, 0.1), maximum depth of each weak learner (3, 5, 8), and minimum loss reduction required to make another partition (0, 1, 5). Then, we ran XGBoost on the training data using squared loss as the objective function. Our response variable and features were the same used in the first-principles energy model. We used the stochastic approach by subsampling 80% of the data at each tree fitting. Our algorithm used up to 1,000 trees; however, if there was no significant increase in performance after 50 trees, the algorithm stopped. Then, we plotted the number of trees (up to 1,000) versus training error and estimated test error via 5-fold cross validation.

After tuning, the model was trained with the optimal hyperparameters on the entire training set, and AREs were computed for the flights on the test set. We selected the hyperparameter combination to be used for each regime (number of trees, learning rate, maximum depth, minimum loss reduction) by choosing a low test error, low generalization error, and low standard errors. The selected hyperparameter values are in Table S1.

Estimation of the standard errors for the coefficients

To obtain standard errors of the estimated coefficients, we used a nonparametric bootstrap approach.[41] 1,000 bootstrap replications were used to resample with replacement the 120 training flights, and the energy models for the three flight regimes were refitted for each bootstrap sample. At the end, the standard errors (SEs) of the coefficients were obtained from their sampling distribution.

Model predictive power

To evaluate the model's predictive power, the regime-specific fitted models were then applied to the testing flights of the test set and were compared by ARE, computed at flight resolution. That is, for each flight from the test set, we computed their $E_{measured}$ integrating power over time and the $E^m_{estimated}$ as the sum of the integral of the estimated power over time for the three flight regimes via method m:

$$ARE(m) = \left| \frac{E_{measured} - E^m_{estimated}}{E_{measured}} \right| \quad \text{(Equation 9)}$$

for $m \in \{EnergyModel, XGBoost\}$.

Figure S12 shows the AREs on the test set for the energy models developed in this study.

Estimation of drone range

Our analysis also shows the impact of varying operational parameters (speed, altitude, and payload) on the range of the drone. The two-way drone range (d) can be calculated considering the cruise speed

$$E = \sum \left(b_1 \frac{m^{1.5}}{\sqrt{\rho}} + b_0 \right) t = \sum E_{takeoff} + \sum E_{landing} + \sum \left(b_1 \frac{m^{1.5}}{\sqrt{\rho}} + b_0 \right) \frac{d}{V_{cr}}. \quad \text{(Equation 10)}$$

Expanding for a two-way trip and solving for d

$$d = \frac{\left[E_{max} - \left(E^l_{takeoff} + E^u_{takeoff} + E^l_{landing} + E^u_{landing} \right) \right] V_{cr}}{b_1 \left(\frac{m^{1.5}}{\sqrt{\rho}}^l + \frac{m^{1.5}}{\sqrt{\rho}}^u \right) + 2b_0}, \quad \text{(Equation 11)}$$

Table 3. Base-case energy consumption and GHG emissions for different vehicles

Vehicle class	Energy consumption (MJ/km)	Fuel GHG emissions (g/km)	Upstream GHG emissions (g/km)	Battery GHG emissions (g/km)	Energy consumption (MJ/package)	GHG emission (g/package)
Medium-duty diesel truck	11.00	762.8	168.7	–	5.24	443.6
Small diesel van	4.90	339.8	75.2	–	1.41	119.2
Medium-duty electric truck	3.80	674.3	83.7	24.5	1.81	372.6
Small electric van	1.65	293.0	36.4	14.1	0.47	98.7
Electric cargo bicycle	0.10	17.9	2.2	3.3	0.10	23.4
Small quadcopter drone	0.08	14.4	1.8	1.3	0.33	70.1

where E_{max} is the energy available in the battery; $E_{takeoff}$ and $E_{landing}$ are the energy consumed during takeoff and landing for delivery (loaded = l) and returning (unloaded = u), respectively; b_1 and b_0 the coefficients from Table 1 for cruise; and V_{cr} is the average inertial cruise speed.

The energy during takeoff and landing can be calculated as

$$E = \left(b_1 \frac{m^{1.5}}{\sqrt{\rho}} + b_0\right) \frac{h}{V}, \quad \text{(Equation 12)}$$

where h is the cruise altitude and V is the average speed during takeoff and landing.

For instance, a small quadcopter operating at V_{cr} = 12 m/s, payload = 500 g, h = 100 m, takeoff average speed (V_{tk}) = 2.5 m/s, and landing average speed V_{ld} = 2.0 m/s has a range of approximately 4 km (2 km of one-way delivery range), consuming approximately 74.5 Wh (per round-trip delivery).

Therefore, a quadcopter drone flying under these conditions would consume approximately 0.067 MJ/km, not considering charging and transmission losses. The energy consumption during takeoff and landing for this trip corresponds to approximately 36% of the total energy consumption (26.8 Wh per trip). This share of energy could be reduced by 95% in a 5-m takeoff (to 1.34 Wh per trip), which could be achieved, for instance, if the drone would depart from the top of a building, provided sufficient space for safe operation. This would reduce total trip energy by 34% (74.5–48.95 Wh) or increase the range from 2 to 3 km (4–6 km two-way distance), consuming the same amount of energy. Further analysis of system-wide energy benefits of building-to-building drone travel is an important topic for future research.

Transport mode comparison

We develop a model comparing the small quadcopter drone with different transportation modes in terms of energy consumption and CO_2e emissions and validate it against top-down sustainability reports from UPS in 2019. The energy consumption of a medium-duty diesel truck is considered as 11 MJ/km[43]. Whereas a medium-duty electric truck has an energy consumption of 1.4 kWh/mile,[42] or 3.13 MJ/km, diesel vans operate at 18.4 MPG on average,[43] or 4.9 MJ/km (conversion factors: one gallon of diesel = 137,381 Btu,[44] 1 MJ = 947.817 Btu, one mile = 1.60934 km). On the other hand, electric vans operate with an energy consumption of 0.38 kWh/km,[45] or 1.36 MJ/km. Finally, an electric cargo bicycle operates at 0.023 kWh/km,[46] or 0.08 MJ/km (conversion factors: 1 kWh = 3.6 MJ). In addition, variations in driving style can vary energy consumption by 40%.[47]

Based on our energy model, a small quadcopter drone consumes approximately 74 Wh in a 2-km delivery distance (4-km total distance), or 0.067 MJ/km, when delivering at maximum capacity (0.5-kg payload with unloaded return) and a cruise speed of 12 m/s. Transmission losses of 6.5% and a charging efficiency of 88%[32,48–50] were included with the energy consumption of the electric vehicles (Table 3). Table S1 summarizes the nominal energy consumption and also provides the payload capacity of each mode.

The electricity CO_2e emissions were considered to be the 2020 American non-baseload average of 177 g/MJ (638 g/kWh), with the lower bound being 111 g/MJ (399 g/kWh) from New England and the upper limit being 250 g/MJ (900 g/kWh), reflecting non-baseload emissions from the central Midwest.[32] CO_2e emissions for diesel combustion was considered as 1.61×10^{-4} lb/Btu,[51] or 69.35 g/MJ. Upstream GHG emissions for average diesel and electricity generation are 15 g/MJ and 22 g/MJ[53], respectively. The battery life cycle emissions for the drone (assumed to be similar to Li-iron phosphate) were calculated as 0.76 (base case), 0.23 (low case), and 1.52 g/km (high case). Similarly, the electric cargo bicycle has battery life cycle emissions of 1.3 g/km, considering a Li-ion NMC811 battery. For the electric van and electric medium-duty truck, we assumed a battery of Li-ion NMC811, resulting in 14.1 g/km for the van and 24.5 g/km for the truck.[52] Energy and emissions associated with system changes beyond the vehicle (such as in warehousing[20]) are not included and are important areas for future work.

The energy consumption per package (E_{pack}) was calculated as

$$E_{pack} = \frac{E_{dist}}{S_{freq} \cdot P_{freq}}, \quad \text{(Equation 13)}$$

where E_{dist} is the energy consumption per distance unit, S_{freq} is the number of stops to deliver packages per distance unit, and P_{freq} is number of packages delivered per stop on average.

Similarly, the GHG emissions per package (GHG_{pack}) is calculated as

$$GHG_{pack} = \frac{E_{dist} \cdot GHG_{energy}}{S_{freq} \cdot P_{freq}}, \quad \text{(Equation 14)}$$

where GHG_{energy} is the mass of CO_2e per energy unit.

For this study, we used the US National Renewable Energy Laboratory's Fleet DNA: Commercial Fleet Operating Data[53] to estimate the number of stops per km of delivery trucks and vans. Therefore, we assumed that trucks and vans have an average of 0.7 and 1.74 stops/km, respectively. In addition, we assumed that trucks and vans would deliver an average of three and two packages per stop, respectively. Similarly, we assumed that an electric cargo bicycle and a small quadcopter drone would experience 1 and 0.25 stops/km, respectively, and deliver one package per stop on average. We also varied these parameters in order to assess their sensitivity in the final outcome. Table S2 provides additional details on the values assumed for delivery intensity. Tables S3 and S4 provide life cycle GHG emissions for vehicle per km and package, respectively, for each US Environmental Protection Agency (EPA) sub-region. Table 3 summarizes the values calculated per vehicle.

SUPPLEMENTAL INFORMATION

Supplemental information can be found online at https://doi.org/10.1016/j.patter.2022.100569.

ACKNOWLEDGMENTS

This work was supported by the US Department of Energy's Vehicle Technologies Office, award number DE-EE0008463. This article was prepared while C.S. and H.M. were with Carnegie Mellon University. The opinions expressed in this article are the authors' own and do not reflect the view of the United States government or any other organization. We are very grateful to the reviewers whose comments have substantially improving the manuscript.

AUTHOR CONTRIBUTIONS

The research project was conceived by C.S., H.S.M., and S.S. The drone data collection experiment was designed by J.P. The energy model was developed by T.A.R. The results were analyzed by T.A.R. and N.L.O. The GHG emissions analysis was performed by T.A.R. and C.S. All authors contributed to the writing of the manuscript.

DECLARATION OF INTERESTS

The authors declare no competing interests. C.S. has a patent for a system, method, and computer program product for transporting an unmanned vehicle, US patent 11300978B1, 2022. The patent's methods were not used this study.

INCLUSION AND DIVERSITY

One or more of the authors of this paper self-identifies as an underrepresented ethnic minority in science. One or more of the authors of this paper self-identifies as a member of the LGBTQ+ community.

Received: December 5, 2021
Revised: May 13, 2022
Accepted: July 13, 2022
Published: August 5, 2022

REFERENCES

1. Davis, S., and Boundy, R. (2021), Edition 39 (Transportation Energy Data Book). https://doi.org/10.2172/1767864.
2. Dost, F., and Maier, E. (2018). E-commerce effects on energy consumption: a multi-year ecosystem-level assessment. J. Ind. Ecol. *22*, 799–812. https://doi.org/10.1111/jiec.12639.
3. Doole, M., Ellerbroek, J., and Hoekstra, J. (2020). Estimation of traffic density from drone-based delivery in very low level urban airspace. J. Air Transport. Manag. *88*, 101862. https://doi.org/10.1016/J.JAIRTRAMAN.2020.101862.
4. Mims, C. (2020). The scramble for delivery robots is on and startups can barely keep up. Wall Street Journal. https://www.wsj.com/articles/the-scramble-for-delivery-robots-is-on-and-startups-can-barely-keep-up-11587787199.
5. Lee, T.B. (2020). The pandemic is bringing us closer to our robot takeout future. ARS Tech. https://arstechnica.com/tech-policy/2020/04/the-pandemic-is-bringing-us-closer-to-our-robot-takeout-future/.
6. Pani, A., Mishra, S., Golias, M., and Figliozzi, M. (2020). Evaluating public acceptance of autonomous delivery robots during COVID-19 pandemic. Transport. Res. Transport Environ. *89*, 102600. https://doi.org/10.1016/j.trd.2020.102600.
7. Kapser, S., and Abdelrahman, M. (2020). Acceptance of autonomous delivery vehicles for last-mile delivery in Germany – extending UTAUT2 with risk perceptions. Transport. Res. C Emerg. Technol. *111*, 210–225. https://doi.org/10.1016/j.trc.2019.12.016.
8. Abrar, M.M., Islam, R., and Shanto, M.A.H. (2020). An autonomous delivery robot to prevent the spread of coronavirus in product delivery system. In 2020 11th IEEE Annual Ubiquitous Computing, Electronics and Mobile Communication Conference, UEMCON 2020 (Institute of Electrical and Electronics Engineers Inc.), pp. 0461–0466. https://doi.org/10.1109/UEMCON51285.2020.9298108.
9. Carter, S., Johnston, T., Lidel, S., Riedel, R., and Tusch, L. (2022). Drone Delivery: More Lift than You Think. McKinsey Co. https://www.mckinsey.com/industries/aerospace-and-defense/our-insights/future-air-mobility-blog/drone-delivery-more-lift-than-you-think.
10. Sheth, M., Butrina, P., Goodchild, A., and McCormack, E. (2019). Measuring delivery route cost trade-offs between electric-assist cargo bicycles and delivery trucks in dense urban areas. Eur. Transp. Res. Rev. *11*, 11–12. https://doi.org/10.1186/s12544-019-0349-5.
11. McLeod, F.N., Cherrett, T.J., Bektas, T., Allen, J., Martinez-Sykora, A., Lamas-Fernandez, C., Bates, O., Cheliotis, K., Friday, A., Piecyk, M., and Wise, S. (2020). Quantifying environmental and financial benefits of using porters and cycle couriers for last-mile parcel delivery. Transport. Res. Transport Environ. *82*, 102311. https://doi.org/10.1016/j.trd.2020.102311.
12. Wagoner, D. (2022). Five Trends Changing the Way Customers Shop in 2022. Forbes. https://www.forbes.com/sites/forbesbusinesscouncil/2022/01/31/five-trends-changing-the-way-customers-shop-in-2022/?sh=2da296854021.
13. Muratori, M., and Mai, T. (2020). The shape of electrified transportation. Environ. Res. Lett. *16*, 011003–019326. https://doi.org/10.1088/1748-9326/ABCB38.
14. Zhang, J., Campbell, J.F., Sweeney II, D.C., and Hupman, A.C. (2021). Energy consumption models for delivery drones: a comparison and assessment. Transport. Res. Transport Environ. *90*, 102668. https://doi.org/10.1016/j.trd.2020.102668.
15. D'Andrea, R. (2014). Guest editorial can drones deliver? IEEE Trans. Autom. Sci. Eng. *11*, 647–648. https://doi.org/10.1109/TASE.2014.2326952.
16. Figliozzi, M.A. (2017). Lifecycle modeling and assessment of unmanned aerial vehicles (Drones) CO2e emissions. Transport. Res. Transport Environ. *57*, 251–261. https://doi.org/10.1016/j.trd.2017.09.011.
17. Dorling, K., Heinrichs, J., Messier, G.G., and Magierowski, S. (2017). Vehicle routing problems for drone delivery. IEEE Trans. Syst. Man, Cybern. Syst. *47*, 70–85. https://doi.org/10.1109/TSMC.2016.2582745.
18. Liu, Z., Sengupta, R., and Kurzhanskiy, A. (2017). A power consumption model for multi-rotor small unmanned aircraft systems. In 2017 International Conference on Unmanned Aircraft Systems (ICUAS) (IEEE), pp. 310–315. https://doi.org/10.1109/ICUAS.2017.7991310.
19. Lohn, A.J., Gulden, T., Xu, J., Jones, T., Kuhn, K., and Welser, W.I. (2018). What the Buzz? The Drones Are Here. Rand Corporation. https://www.rand.org/pubs/research_briefs/RB9995.html.
20. Stolaroff, J.K., Samaras, C., O'Neill, E.R., Lubers, A., Mitchell, A.S., and Ceperley, D. (2018). Energy use and life cycle greenhouse gas emissions of drones for commercial package delivery. Nat. Commun. *9*, 409. https://doi.org/10.1038/s41467-017-02411-5.
21. Jeong, H.Y., Song, B.D., and Lee, S. (2019). Truck-drone hybrid delivery routing: payload-energy dependency and No-Fly zones. Int. J. Prod. Econ. *214*, 220–233.
22. Torabbeigi, M., Lim, G.J., and Kim, S.J. (2019). Drone delivery scheduling optimization considering payload-induced battery consumption rates. J. Intell. Rob. Syst. *97*, 471–487. https://doi.org/10.1007/s10846-019-01034-w.
23. Kirschstein, T. (2020). Comparison of energy demands of drone-based and ground-based parcel delivery services. Transport. Res. Transport Environ. *78*, 102209. https://doi.org/10.1016/j.trd.2019.102209.
24. Alyassi, R., Khonji, M., Karapetyan, A., Chau, C.-K., Elbassioni, K., and Tseng, C.-M. (2017). Autonomous Recharging and Flight Mission Planning for Battery-Operated Autonomous Drones. In IEEE Transactions on Automation Science and Engineering (IEEE). https://doi.org/10.1109/TASE.2022.3175565.
25. Figliozzi, M.A. (2020). Carbon emissions reductions in last mile and grocery deliveries utilizing air and ground autonomous vehicles. Transp. Res. D Transp. Environ. *85*, 102443. https://doi.org/10.1016/j.trd.2020.102443.
26. Goodchild, A., and Toy, J. (2018). Delivery by drone: an evaluation of unmanned aerial vehicle technology in reducing CO2 emissions in the delivery service industry. Transport. Res. Transport Environ. *61*, 58–67. https://doi.org/10.1016/j.trd.2017.02.017.
27. Borlaug, B., Muratori, M., Gilleran, M., Woody, D., Muston, W., Canada, T., Ingram, A., Gresham, H., and McQueen, C. (2021). Heavy-duty truck electrification and the impacts of depot charging on electricity distribution

systems. Nat. Energy *6*, 673–682. https://doi.org/10.1038/s41560-021-00855-0.

28. Speedbird Aero. Meet our aircraft. https://www.speedbird.aero/index-en.html#aircraft.
29. Manna Drone Delivery. Introducing Manna. Drone delievery made simple. https://www.manna.aero/.
30. Hamilton, I.A. (2020). A tiny Irish drone startup is delivering emergency cake, pizza, and medical supplies to Moneygall, the ancestral home of Barack Obama. Business Insider. https://www.businessinsider.com/drone-delivery-startup-manna-adapted-to-coronavirus-pandemic-2020-5.
31. Rodrigues, T.A., Patrikar, J., Choudhry, A., Feldgoise, J., Gahlaut, A., Arcot, V., Lau, S., Moon, B., Wagner, B., Matthews, H.S., et al. (2021). In-flight positional and energy use data set of a DJI Matrice 100 quadcopter for small package delivery. Sci. Data *8*, 1–8. https://doi.org/10.1038/s41597- 430 021-00930-x.
32. United States Environmental Protection Agency (2015). Emissions & Generation Resource Integrated Database (eGRID). https://www.epa.gov/egrid.
33. Federal Aviation Administration (2020). Small Unmanned Aircraft Systems (UAS) Regulations (Part 107). https://www.faa.gov/newsroom/small-unmanned-aircraft-systems-uas-regulations-part-107.
34. Elsayed, M., and Mohamed, M. (2020). The impact of airspace regulations on unmanned aerial vehicles in last-mile operation. Transport. Res. Transport Environ. *87*, 102480. https://doi.org/10.1016/j.trd.2020.102480.
35. UPS. 2019 GRI Content Index. https://about.ups.com/content/dam/upsstories/assets/reporting/2019-UPS-Corporate-Global-Reporting-Initiative-GRI-Content-Index.pdf.
36. Isik, M., Dodder, R., and Kaplan, P.O. (2021). Transportation emissions scenarios for New York City under different carbon intensities of electricity and electric vehicle adoption rates. Nat. Energy *6*, 92–104. https://doi.org/10.1038/s41560-020-00740-2.
37. Rodrigues, T.A., Patrikar, J., Choudhry, A., Feldgoise, J., Arcot, V., Gahlaut, A., Lau, S., Moon, B., Wagner, B., Matthews, H.S., et al. (2020). Data collected with package delivery quadcopter drone. figshare. https://doi.org/10.1184/R1/12683453.
38. ROS (2010). message_filters::sync::ApproximateTime. http://wiki.ros.org/message_filters/ApproximateTime.
39. Rotaru, C., and Todorov, M. (2017). Helicopter flight physics. In Flight Physics - Models, Techniques and Technologies (InTech). https://doi.org/10.5772/intechopen.71516.
40. Chen, T., He, T., Benesty, M., Khotilovich, V., Tang, Y., Cho, H., Chen, K., Mitchell, R., Cano, I., Zhou, T., et al. (2020). Xgboost: Extreme Gradient Boosting. https://github.com/dmlc/xgboost.
41. Efron, B., and Tibshirani, R.J. (1993). An Introduction to the Bootstrap (Chapman & Hall/CRC).
42. Prohaska, R., Simpson, M., Ragatz, A., Kelly, K., Smith, K., and Walkowicz, K. (2016). Field Evaluation of Medium-Duty Plug-In Electric Delivery Trucks (US Department of Energy). https://doi.org/10.2172/1337010.
43. Advantage Outfitters. Best Cargo Vans for Small Businesses: Gas Mileage, Cargo Space and More. https://www.commercialvanshelving.com/blog/best-cargo-van-small-businesses.
44. U.S. Energy Information Administration (EIA). Units and calculators explained. https://www.eia.gov/energyexplained/units-and-calculators/.
45. Fiori, C., and Marzano, V. (2018). Modelling energy consumption of electric freight vehicles in urban pickup/delivery operations: analysis and estimation on a real-world dataset. Transport. Res. Transport Environ. *65*, 658–673. https://doi.org/10.1016/j.trd.2018.09.020.
46. Erlandsson, J. (2017). The Armadillo Electric Cargo Bike Use 6 % of the Electricity of a Small Electric Van. Velove. https://www.velove.se/news/armadillo-cargo-bike-use-6-electricity-small-electric-van.
47. U.S. Department of Energy. Many Factors Affect MPG. https://www.fueleconomy.gov/feg/factors.shtml#:~:text=Aggressive%20driving%20(speeding%2C%20rapid%20acceleration,more%20idling%20will%20lower%20MPG.
48. Elgowainy, A., Han, J., Ward, J., Joseck, F., Gohlke, D., Lindauer, A., Ramsden, T., Biddy, M., Alexander, M., Barnhart, S., et al. (2016). Cradle-to-Grave Lifecycle Analysis of U.S. Light-Duty Vehicle-Fuel Pathways: A Greenhouse Gas Emissions and Economic Assessment of Current (2015) and Future (2025-2030) Technologies (Argonne National Laboratory).
49. Shiau, C.S.N., Samaras, C., Hauffe, R., and Michalek, J.J. (2009). Impact of battery weight and charging patterns on the economic and environmental benefits of plug-in hybrid vehicles. Energy Pol. *37*, 2653–2663.
50. U.S. Energy Information Administration (EIA) (2022). How much carbon dioxide is produced per kilowatthour of U.S. electricity generation?. https://www.eia.gov/tools/faqs/faq.php?id=74&t=11.
51. U.S. Energy Information Administration (EIA) (2022). How much carbon dioxide is produced when different fuels are burned?. https://www.eia.gov/tools/faqs/faq.php?id=73&t=11.
52. Argonne National Laboratory (2020). GREET. https://greet.es.anl.gov/.
53. National Renewable Energy Laboratory. Fleet DNA: Commercial Fleet Vehicle OperatinData. https://www.nrel.gov/transportation/fleettest-fleet-dna.html.

Patterns, Volume *3*

Supplemental information

Drone flight data reveal energy and greenhouse gas emissions savings for very small package delivery

Thiago A. Rodrigues, Jay Patrikar, Natalia L. Oliveira, H. Scott Matthews, Sebastian Scherer, and Constantine Samaras

Supplemental Experimental Procedures

S1. Per ton-km comparison

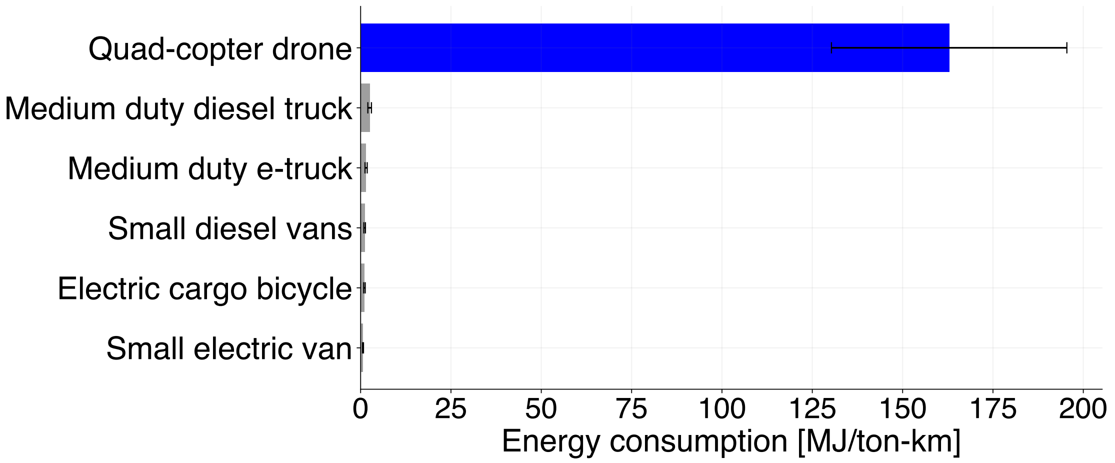

Figure S1: Energy consumption comparison on a per ton-km basis. Error bars represent variations in driving styles and vehicle characteristics

S2. Delivery intensity to match drone's GHG emissions per package

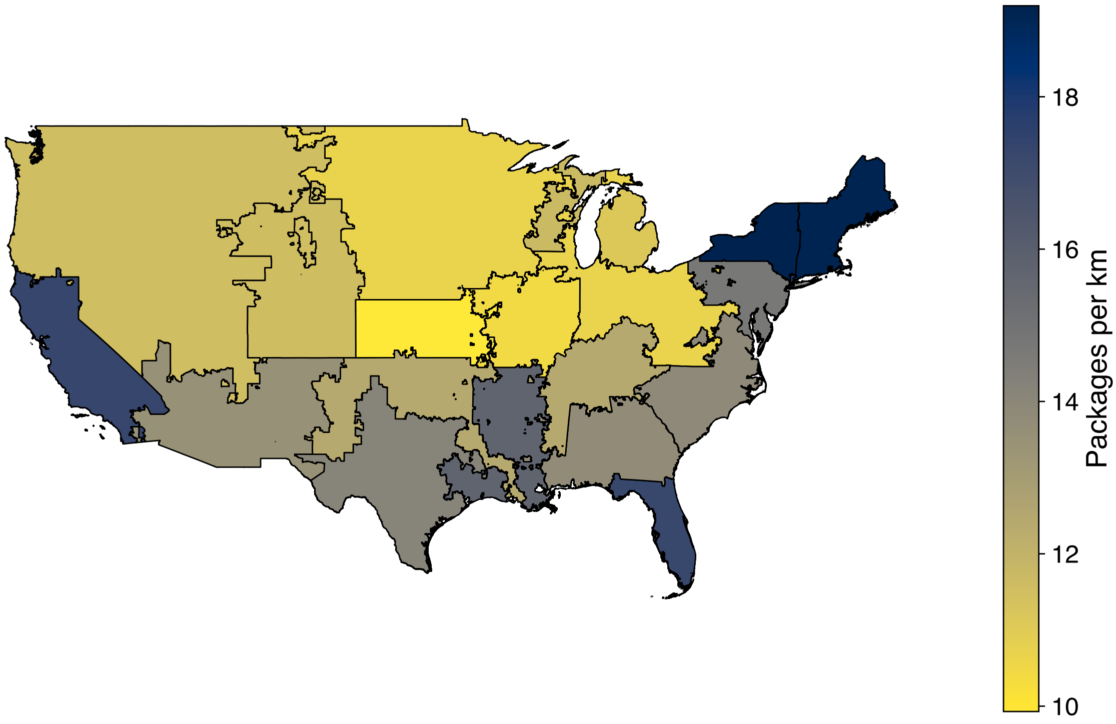

Figure S2: Medium duty diesel truck delivery intensity in packages per km to meet drone's GHG emission per sub-region of the US. Our base model assumes a drone delivering 0.25 packages per km, i.e. a two-way delivery distance of 4 km, consuming 0.27 MJ/package.

S3. Defining flight regimes

An example of the drone's altitude versus time (Figure S3) shows very clearly when the three flight regimes (take off, cruise, and landing) start and end. The steady climbing movement in the first part of the flight rep-

resents the Take Off regime. The relative constant altitude between 50 and 170 seconds correspond to the Cruise regime. Lastly, the steady descent movement at the end of the flight represents the Landing regime.

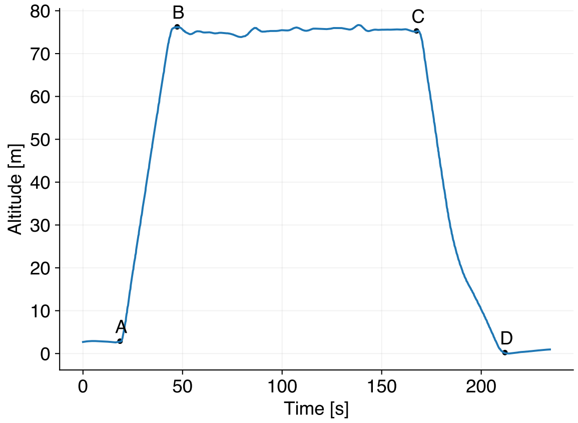

Figure S3: Altitude over time. Points **A**, **B**, **C**, and **D** indicate the starting and ending points for each flight regime. Takeoff, cruise and landing regimes are represented between points **AB**, **BC**, and **CD**, respectively.

Although defining the interval where the three regimes occur seems to be quite intuitive for human eyes, the inherent variability of the data provided by the GPS make the process less efficient for an algorithm. Therefore, a Gaussian Filter was applied to the data in order to reduce eventual noises. A sigma of 5 was used, as it approximately reflects the accuracy of the GPS measurements. The result shown in Figure S4 is a much smoother curve.

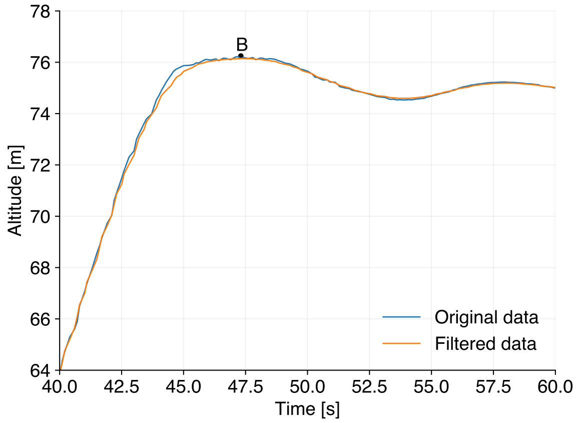

Figure S4: Gaussian filter applied to a portion of the altitude data. Blue curve curve represents the original data and the smoother red curve represents the filtered data. Point **B** indicates the end of the takeoff regime.

Once the Gaussian filter has been applied to the altitude curve, the first derivative provides the points of maximum and minimum altitude variations (Figure S5) that will be used to define the beginning and ending of each regime.

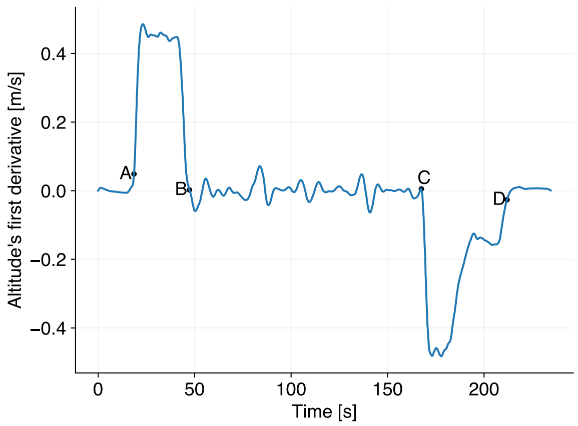

Figure S5: First derivative of the altitude over time. Points **A**, **B**, **C**, and **D** indicate the starting and ending points of the flight regimes. Takeoff, cruise and landing regimes are represented between points **AB**, **BC**, and **CD**, respectively.

It has been established that the Take Off regime would start when the slope reached 0.05 for the first time (**A**) and it would end once the slope reached zero, after reaching maximum slope point (**B**). Similarly, Landing was defined to begin at the first time the slope reached 0 moving backwards from the minimum slope point (**C**) and ends when the slope would reach negative -0.25 moving forward from the minimum slope point (**D**). The Cruise regime is defined as the interval between **B** and **C**.

The result allows the automatic identification of the time interval where the three flight regimes occur throughout the flights. Figure S6 shows the outcome of a particular flight, divided into three regimes.

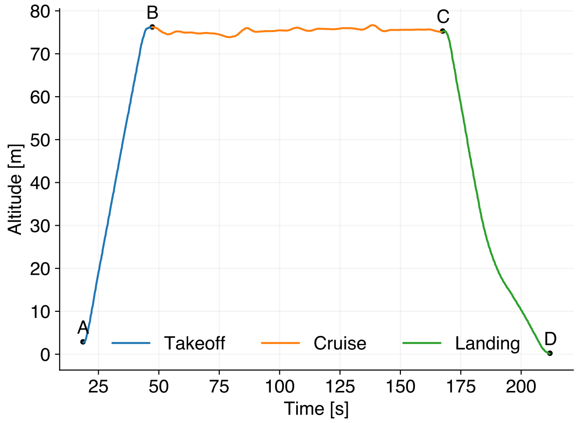

Figure S6: Flight with flight regimes defined. Takeoff, cruise and landing regimes are represented between points **AB**, **BC**, and **CD**, respectively.

The script that automates the flight regime classification is available at https://doi.org/10.5281/zenodo.6726991.

S4. Coordinate Frames

The energy model developed uses a first-principle analysis based on helicopter aerodynamics,[1] which defines different coordinate frames for an aircraft. All the components were considered regarding the drone's body frame as described below.

Different coordinate frames can be used to understand the dynamic behavior of a Unmanned Aerial Vehicle (UAVs).[2] Here we will use the inertial frame, vehicle frame, body frame, stability frame and wind frame, to be defined as follows.

The *inertial frame* has a fixed latitude-longitude origin and uses the North-East coordinates as **i** and **j** directions and the **k** axis towards the center of the Earth (Figure S7a[1]) . Similarly, the *vehicle frame* uses the same north-east-down (NED) directions, but with its origin fixed on the UAV's center of mass (G) (Figure S7b).

[1]CAD Model adapted from[3]

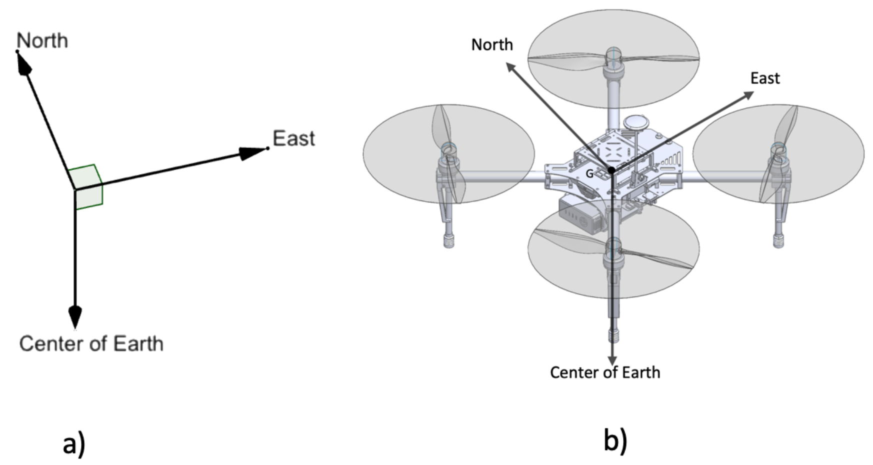

Figure S7: Inertial and Vehicle Frames

The *body frame*, on the other hand, considers the **i**, **j** and **k** directions regarding the body of the aircraft. For instance, the x-axis was defined as the direction along the front of the aircraft and the mass center of the drone (Matrice 100). The angle between the i^v (North) and i^b on the i^vGj^v plane is defined as ψ (Figure S8a). The angle between i^b and i^v on the i^vGk^v plane is defined as θ (Figure S8b). Finally, the angle between j^v and j^b on the j^vGk^v plane is defined as ϕ (Figure S8c).

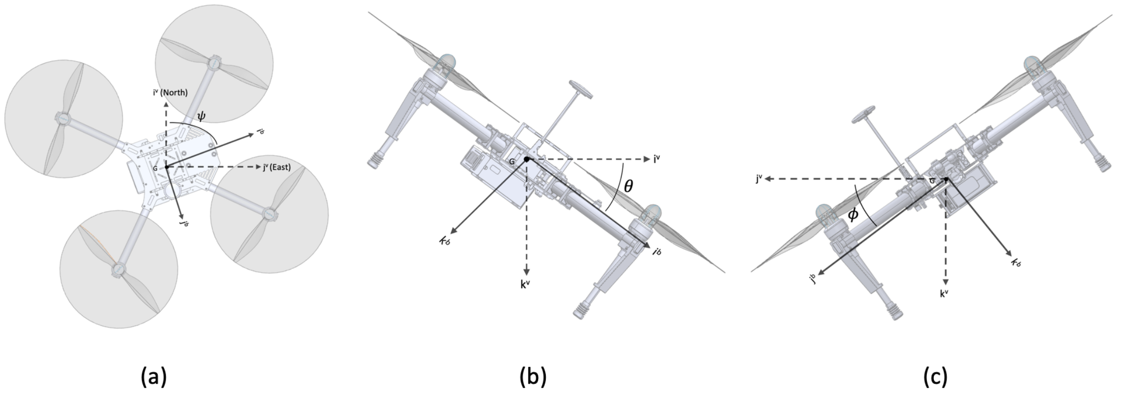

Figure S8: Body Frame

The *stability frame* correspond to the frame of air surrounding the aircraft. The airspeed vector (V_a), defined as the difference between the ground velocity (V_g) and the wind velocity (V_w), flows through the aircraft at a i^sGj^s plane, that is rotated from the body frame reference by α, the angle of attack. Finally, the *wind frame* assumes the **i** axis in the same direction of the airspeed vector. The angle between i^s and V_a is defined as the side slip angle (β) (Figure S9).

With the main working frames described, it is possible to transcribe any vector written as **A** = $a_x\mathbf{i}+a_y\mathbf{j}+a_z\mathbf{k}$ from frame F^0 to frame F^1 using a rotation matrix (R_0^1),

$$\mathbf{A^1} = \left[R_0^1\right] \mathbf{A^0}. \tag{1}$$

For this work, we have established the body frame as the standard working frame. Therefore, all vectors were transcribed to the body frame using the following rotation matrices.[2]

From the vehicle frame to the body frame (R_v^b) the rotation matrix is given as

$$\left[R_v^b\right] = \begin{bmatrix} c_\theta c_\psi & c_\theta s_\psi & -s_\theta \\ s_\phi s_\theta c_\psi - c_\phi s_\psi & s_\phi s_\theta s_\psi + c_\phi c_\psi & s_\phi c_\theta \\ c_\phi s_\theta c_\psi + s_\phi s_\psi & c_\phi s_\theta s_\psi - s_\phi s_\psi & c_\phi c_\theta \end{bmatrix}, \tag{2}$$

where c$_\theta$, c$_\psi$ and c$_\phi$ are the cosines of the angles θ, ψ and ϕ, respectively, and s$_\theta$, s$_\psi$ and s$_\phi$ are the sines of the angles θ, ψ and ϕ, respectively.

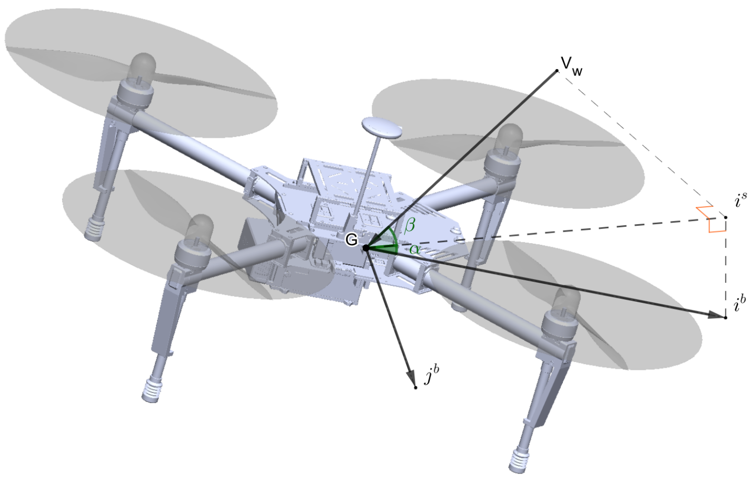

Figure S9: Stability and Wind Frame

The transformation from the stability frame to the body frame is given by the matrix R_s^b

$$[R_s^b] = \begin{bmatrix} \cos(\alpha) & 0 & -\sin(\alpha) \\ 0 & 1 & 0 \\ \sin(\alpha) & 0 & \cos(\alpha) \end{bmatrix}, \tag{3}$$

and the transformation from wind frame to the body frame is given by the matrix R_w^b

$$[R_w^b] = \begin{bmatrix} \cos(\beta)\cos(\alpha) & -\sin(\beta)\cos(\alpha) & -\sin(\alpha) \\ \sin(\beta) & \cos(\beta) & 0 \\ \cos(\beta)\sin(\alpha) & -\sin(\beta)\sin(\alpha) & \cos(\alpha) \end{bmatrix}. \tag{4}$$

S5. First Principles Analysis

The power required for a UAV to maintain a hover in a no-wind condition can be estimated using a first principle analysis established for helicopter aerodynamics. In this case, the power required by a drone can be divided into 4 forms of power: induced power (P_i), profile power (P_p), parasitic power (P_d) and ancillary power (P_a), as described further below.

$$P = P_i + P_p + P_d + P_a \tag{5}$$

S5.1 Induced Power

The induced power (P_i) represents the power required to overcome the force of gravity in order to keep the aircraft in the air, and it can vary according to the flight maneuver.[1] In order to calculate P_i it is necessary to understand how thrust (T) is estimated for a drone propeller. Most of the current models provide thrust from a two-dimension approach, where β = 0 (Figure S10), so that T is given as

$$\mathbf{T} = 2\rho A \mathbf{v_i} \sqrt{(V\cos(\alpha)^2 + (V\sin(\alpha) + v_i)^2}, \tag{6}$$

where ρ represents air density, $\mathbf{v_i}$ induced velocity, V magnitude of the air velocity, and A total area covered by the propellers, calculated by

$$A = M\pi R^2, \tag{7}$$

where M is the number of propellers and R the radius of the propeller.

The induced velocity from a 2D perspective can be estimated through the implicit equation

$$\mathbf{v_i} = \frac{\mathbf{T}}{2\rho A\sqrt{(V\cos(\alpha))^2 + (V\sin(\alpha) + v_i)^2}}. \tag{8}$$

During hover in a no-wind situation, $\alpha = 90^\circ$ and $V = 0$, Equation (8) is reduced to

$$\mathbf{v_i} = \sqrt{\frac{\mathbf{T}}{2\rho A}} \tag{9}$$

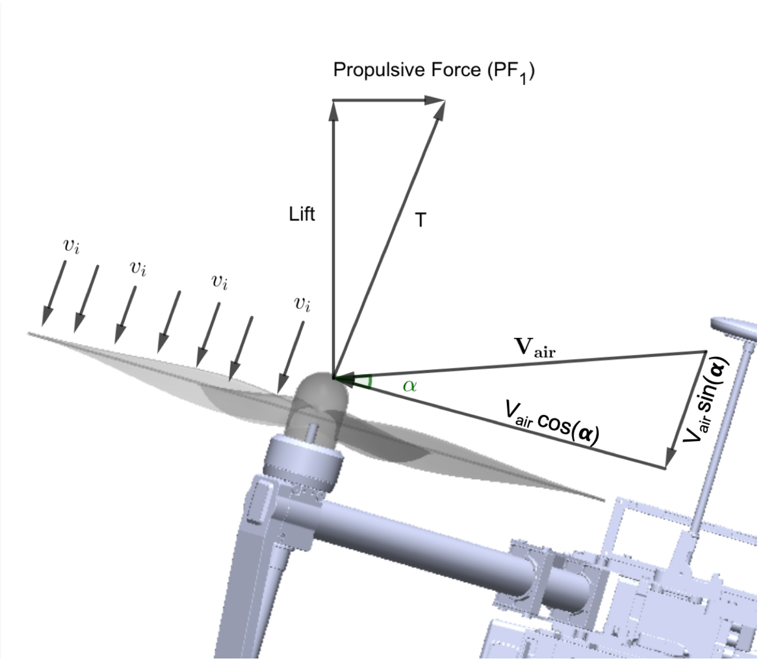

Figure S10: Thrust, β = 0

and $\mathbf{T}$ can be approximated to the drone's weight:

$$T = mg \tag{10}$$

where m represents mass and g gravitational acceleration. Therefore, the induced power during hover in a no-wind condition is calculated as

$$P_i = \mathbf{T} \cdot \mathbf{v_i} = \frac{(mg)^{\frac{3}{2}}}{\sqrt{2\rho A}}. \tag{11}$$

From a spatial perspective **T** must be recalculated to account for $\beta > 0$. Using the same approach used by,[1] and adding β to the relative position of the air flowing through the aircraft, **T** becomes part of a three-dimension force system that accounts for 2 propulsive forces: PF_1 and PF_2 (Figure S11).

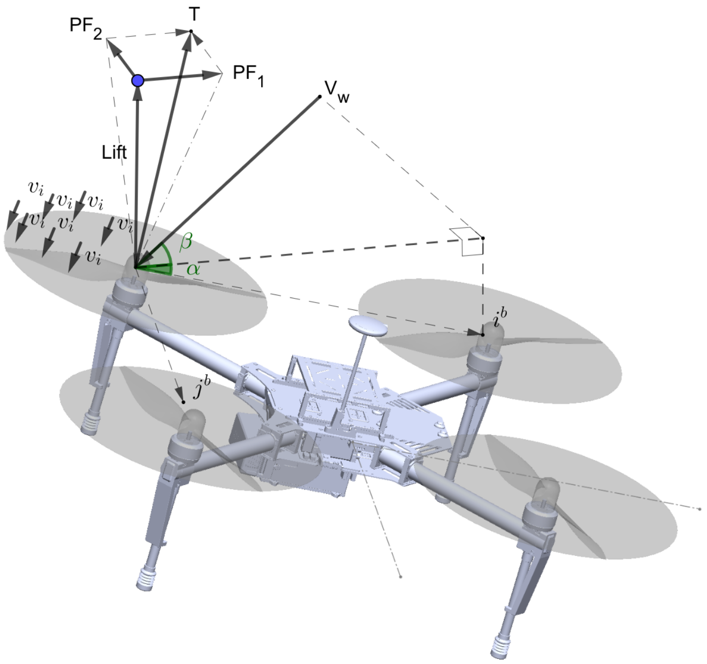

Figure S11: Thrust, $\beta > 0$

$$T = 2\rho A v_i \sqrt{\mathbf{V_i}^2 + \mathbf{V_j}^2 + (\mathbf{V_k} + \mathbf{v_i})^2} \tag{12}$$

where $\mathbf{V_k}$ represents the airspeed vector flowing perpendicularly to the propeller, and $\mathbf{V_i}$ and $\mathbf{V_j}$ are the components acting on the propellers plane. They can be written as a function of $||\mathbf{V}||$ or V, α and β:

$$V_i = V\sqrt{\frac{\sin^2(\alpha)\sin^2(\beta) - \sin^2(\beta)}{\sin^2(\alpha)\sin^2(\beta) - 1}} \tag{13}$$

$$V_j = V\frac{\cos(\alpha)\cos(\beta)}{\sqrt{\sin^2(\alpha)\cos^2(\beta) + \cos^2(\alpha)}} \tag{14}$$

$$V_k = V\sqrt{\frac{\sin^2(\alpha)\sin^2(\beta) - \sin^2(\alpha)}{\sin^2(\alpha)\sin^2(\beta) - 1}} \tag{15}$$

Therefore, P_i for a moving forward flight regime is

$$P_i = T\left(V\sqrt{\frac{\sin^2(\alpha)\sin^2(\beta) - \sin^2(\alpha)}{\sin^2(\alpha)\sin^2(\beta) - 1}} + v_i\right). \tag{16}$$

T can be calculated as a function of θ, ϕ and the rotor lift (L), which in turn can be found as a function of the drone's weight (mg) and the angles θ and ϕ:

$$T = mg\sqrt{\frac{\sin^2(\phi)\cos^2(\theta) + \cos^2(\phi)}{\cos^2(\phi)\cos^2(\theta)}} \tag{17}$$

and from Equation (9), v_i can be calculated through the implicit equation

$$v_i = \frac{\frac{T}{2\rho A}}{\sqrt{V_i^2 + V_j^2 + (V_k + v_i)^2}}. \tag{18}$$

S5.2 Profile Power

The profile power (P_P) represents the power necessary to overcome the drag from the rotating propeller blades.[4] P_p for a hover regime is calculate as

$$P_{p,hover} = \frac{Ncc_{d_{blade}}\rho R^4}{8}\psi\Omega^3 \tag{19}$$

where N is the number of blades in a single propeller, c represents the blade chord width, $c_{d_{blade}}$ is the drag coefficient of the blade, and $\psi\Omega$ is the angular speed.

For a horizontal flight, P_p becomes:

$$P_p = P_{p,hover}(1 + \mu^2) \tag{20}$$

where, μ is the advance ratio for the propeller;

$$\mu = \frac{\sqrt{V_i^2 + V_j^2}}{R\Omega} \tag{21}$$

Assuming that thrust is linearly proportional to the angular speed squared ($T = k\Omega^2$), where k is a scaling factor converting rotor angular speed to thrust, the total profile power is

$$P_P = \sum_{i=1}^{M}\left(\frac{Ncc_{d_{blade}}\rho R^4}{8}\Omega^3(1 + \mu^2)\right). \tag{22}$$

Let c_2 be $\frac{Ncc_{d_{blade}}\rho R^4}{8k^{3/2}}$ and c_3 be $\frac{Ncc_{d_{blade}}\rho R^2}{8k^{1/2}}$, then P_p can be written as

$$P_P = c_2 T^{3/2} + c_3\left(V_i^2 + V_j^2\right)T^{1/2}. \tag{23}$$

S5.3 Parasitic Power

The parasitic power (P_d) is the power required to overcome the drag force resulting from the movement of the quad-copter body through the air.

$$P_d = \sum F^b_{drag} V^b \tag{24}$$

where F^b_{drag} is the drag force on the quad-copter body, and V^b is the airspeed vector acting on the body frame.

$$F^b_{drag} = \frac{1}{2} C^b_d \rho A^b_{ref} (V^b)^2 \tag{25}$$

where C^b_d is the drag coefficient of the drone's body and A^b_{ref} is the reference area.
Therefore,

$$P_d = \sum \frac{1}{2} C^b_d \rho A^b_{ref} (V^b)^3 \tag{26}$$

and is the sum of the parasitic power of all surfaces, P_{di}, P_{dj} and P_{dk}.

S5.4 Ancillary Power

The ancillary power (P_a) is the power required to run all the electronic devices and sensors used for the flight navigation systems and data collection.

Although P_a may vary under extreme operation conditions, such as extremely high and/or low temperatures, it has been assumed that P_a will be relatively constant throughout the flight as long as the number and specifications of the electronic devices and sensors remain the same. Moreover, our data shows that the ancillary power varied between 4 W and 9 W, which corresponds to 1% to 2% of the average power of a flight with vertical movement (approximately 400 W).

S6. Quadratic Programming

Quadratic program (QP) was used to estimate coefficients c_1 to c_6 through the following form[2]

$$\min_x \frac{1}{2} x^T P x + q^T x \tag{27}$$

$$\text{subject to } \begin{bmatrix} Gx \leq h \\ Ax = b \end{bmatrix} \tag{28}$$

Let M be the matrix formed by our B values:

$$M = \begin{bmatrix} B_{11} & B_{21} & B_{31} & B_{41} & B_{51} & B_{61} & 1 \\ B_{12} & B_{22} & B_{32} & B_{42} & B_{52} & B_{62} & 1 \\ \vdots & \vdots & \vdots & \vdots & \vdots & \vdots & \vdots \\ B_{1n} & B_{2n} & B_{3n} & B_{4n} & B_{5n} & B_{6n} & 1 \end{bmatrix}$$

and x be the column matrix formed by our C values:

$$x = \begin{bmatrix} c_1 \\ c_2 \\ \vdots \\ c_7 \end{bmatrix}$$

The estimated power can be then calculated by multiplying Mx

$$P_{estimated} = M.x = \begin{bmatrix} B_{11} & B_{21} & B_{31} & B_{41} & B_{51} & B_{61} & 1 \\ B_{12} & B_{22} & B_{32} & B_{42} & B_{52} & B_{62} & 1 \\ \vdots & \vdots & \vdots & \vdots & \vdots & \vdots & \vdots \\ B_{1n} & B_{2n} & B_{3n} & B_{4n} & B_{5n} & B_{6n} & 1 \end{bmatrix} \begin{bmatrix} c_1 \\ c_2 \\ \vdots \\ c_7 \end{bmatrix}$$

[2] The approach used to solve the quadratic program followed the examples provided at https://scaron.info/blog/quadratic-programming-in-python.html

Let the measured power can be written as:

$$P_{measured} = \begin{bmatrix} P_1 \\ P_2 \\ \vdots \\ P_n \end{bmatrix}$$

The main objective function of the optimization problem is to minimize:

$$minimize \left\| \begin{bmatrix} B_{11} & B_{21} & B_{31} & B_{41} & B_{51} & B_{61} & 1 \\ B_{12} & B_{22} & B_{32} & B_{42} & B_{52} & B_{62} & 1 \\ \vdots & \vdots & \vdots & \vdots & \vdots & \vdots & \vdots \\ B_{1n} & B_{2n} & B_{3n} & B_{4n} & B_{5n} & B_{6n} & 1 \end{bmatrix} \begin{bmatrix} c_1 \\ c_2 \\ \vdots \\ c_6 \end{bmatrix} - \begin{bmatrix} P_1 \\ P_2 \\ \vdots \\ P_n \end{bmatrix} \right\|^2$$

The parameters in x must be positive, so it is necessary to add a constrain:

$$subject\ to \begin{bmatrix} -1 & 0 & 0 & 0 & 0 & 0 & 0 \\ 0 & -1 & 0 & 0 & 0 & 0 & 0 \\ 0 & 0 & -1 & 0 & 0 & 0 & 0 \\ 0 & 0 & 0 & -1 & 0 & 0 & 0 \\ 0 & 0 & 0 & 0 & -1 & 0 & 0 \\ 0 & 0 & 0 & 0 & 0 & -1 & 0 \\ 0 & 0 & 0 & 0 & 0 & 0 & -1 \end{bmatrix} \begin{bmatrix} c_1 \\ c_2 \\ c_3 \\ c_4 \\ c_5 \\ c_6 \\ c_7 \end{bmatrix} \leq \begin{bmatrix} 0 \\ 0 \\ 0 \\ 0 \\ 0 \\ 0 \\ 0 \end{bmatrix}$$

In other words, the optimization problem can be written as:

$$minimize\ ||Mx - b||^2$$

$$subject\ to : [Gx \leq h]$$

where G is a 7x7 identity matrix multiplied by -1, and h is a 7x1 zero matrix.
$||Mx - b||^2$, where b = $P_{measured}$, can be expanded as:

$$\begin{aligned} ||Mx - P||^2 &= (Mx - b)^T (Mx - b) \\ &= x^T M^T Mx - x^T M^T b - b^T Mx + b^T b \\ &\propto \left(\frac{1}{2}\right) x^T M^T Mx - \left(\frac{1}{2}\right) x^T M^T b - \left(\frac{1}{2}\right) b^T Mx \\ &= \left(\frac{1}{2}\right) x^T (M^T M) x + (-M^T b)^T x \end{aligned}$$

Which enables us to write the optimization problem in the quadratic standard, with $P = (M^T M)$, $q = (-M^T b)$, $G = -[I]_{7x7}$ and $h = [0]_{7x1}$

S7. Linear Regression

Here we describe the steps used to perform the linear regression and calculate the coefficients b_1, b_2, and R^2[5] used in the main method.

Let $x = \frac{m^{1.5}}{\sqrt{\rho}}$ and $y = \bar{P}$, where m is the total mass of the drone (including payload), ρ is the air density and $\bar{P}$ is the average power observed during a flight regime.

$$\bar{x} = \frac{\sum_{i=1}^{n} x_i}{n}$$

$$\bar{y} = \frac{\sum_{i=1}^{n} y_i}{n}$$

The sum of squares (S_{xx}) and cross-products (S_{xy}) are calculated as

$$S_{xx} = \sum_{i=1}^{n} (x_i - \bar{x})^2$$

$$S_{xy} = \sum_{i=1}^{n} (x_i - \bar{x})(y_i - \bar{y})$$

The linear coefficients b_1 and b_2 are then calculated as

$$b_1 = \frac{S_{xy}}{S_{xx}}$$

$$b_0 = \bar{y} - b_1\bar{x}$$

The total sum of squares (SS_{tot}) and the regression sum of squares (SS_{reg}) are calculated as

$$SS_{tot} = \sum_{i=1}^{n} (y_i - \bar{y})^2$$

$$SS_{reg} = b_1^2 S_{xx}$$

Finally, the coefficient of determination (R^2) is obtained as

$$R^2 = \frac{SS_{ref}}{SS_{tot}}$$

S8. Machine Learning approach

While regression trees are simple and interpretable, they do not have good generalization properties: if the tree is too deep, it overfits to training data, and if it is too shallow, it does not capture complex structures. Boosting is an ensemble technique that sequentially fits weak regression trees, creating a powerful learner. Each new weak learner tries to correct the errors of the previous learners by targeting the residuals of prior models. `XGBoost` is a library that optimizes speed and performance of gradient boosted trees algorithms. In addition to the advantages of the gradient boosting algorithm, `XGBoost` also adds useful features that the user can control when running the model, such as selecting the learning rate, using a stochastic approach via subsampling, and adding regularization to improve generalization.

We use `XGBoost` as a gold standard when evaluating the energy model performance. Our modeling approach via `XGBoost` consists of the following steps:

1. Fix a regime and a set of hyperparameter values to be tuned. We varied the following hyperparameters: learning rate (0.01, 0.05, 0.1), maximum depth of each weak learner (3, 5, 8), minimum loss reduction required to make another partition (0, 1, 5).
   (a) Run `XGBoost` on the training data, using squared loss as the objective function. Our response variable and features are the same used in the first principles energy model. We used the stochastic approach by subsampling 80% of the data at each tree fitting. Our algorithm used up to 1000 trees; however, if there was no significant increase in performance after 50 trees, the algorithm stopped. Test error estimation for hyperparameter tuning was performed via 5-fold cross validation.
   (b) Plot number of trees (up to 1000) versus training error and estimated test error via 5-fold cross validation.
2. Select hyperparameter combination to be used for each regime (number of trees, learning rate, maximum depth, minimum loss reduction) by choosing a low test error, low generalization error[3], and low standard errors. The selected hyperparameter values are in Table S1.
3. Fit selected model for each regime using training data and evaluate its performance by computing errors (ARE) using test data.

	Take Off	Cruise	Landing
learning rate	0.05	0.1	0.1
maximum depth	3	3	3
minimum loss reduction	0	5	5
number of trees	250	200	250

Table S1: Selected hyperparameter values for each flight regime.

[3] Generalization error is the difference between training error and test error.

S9. Prediction power of the energy model

Figure S12 presents the Absolute Relative Error on the test set for the three models. Model 1 is described in the main paper. Model 2 corresponds to a model including the four forms of power described in equation 5 of the section “First Principles Analysis” of this Supplementary Information. Between the two proposed models, Method 1 showed considerably lower average relative error of 2.1% when compared to Method 2 (with average relative error of 10%). Given its high flexibility and working as our optimal reference for predictive power, `XGBoost` attained the lowest average relative error (2%) as expected. Method 1’s absolute relative error is fairly close to `XGBoost`, supporting Method 1 as a good model both for predictive power and interpretability.

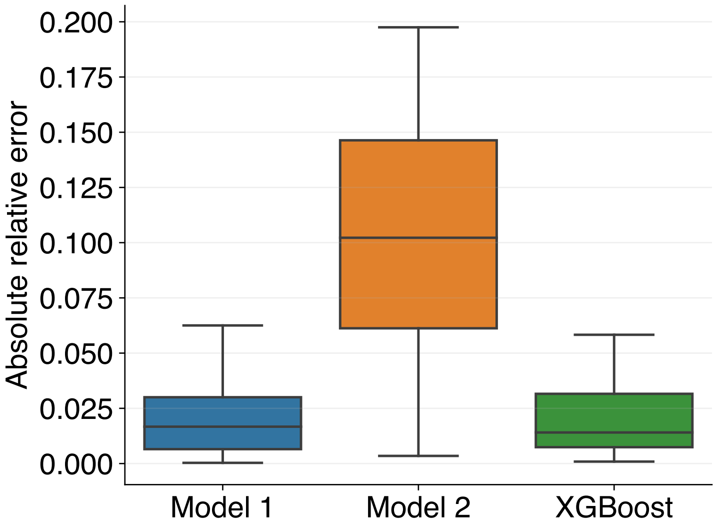

Figure S12: Boxplots of Absolute Relative Error (ARE) computed based on the test set of flights using Method 1, Method 2, and `XGBoost`.

S10. Parameters used in the sensitivity analysis

Table S2 provides the delivery intensity values assumed for lower, base and upper cases.

Table S2: Values assumed for delivery intensity for lower, base, and upper cases.

**Vehicle Class**	**Delievery intensity [packages/km] (lower, base, upper)**
Medium duty truck	(1.5, 2.1, 5)
Small diesel van	(1.5, 3.5, 5)
Medium duty electric truck	(1.5, 2.1, 5)
Small electric van	(1.5, 3.5, 5)
Electric cargo bicycle	(0.25, 1, 3)
Quad-copter drone	(0.125, 0.25, 0.5)

Table S3 and Table S4 provide the GHG emissions per km and per package, respectively, of the electric vehicles assessed in this study.

Table S3: Life cycle GHG emissions by vehicle per km for each EPA subregion [gCO_2e/km]. Direct emissions from EPA Subregions,[6] upstream electricity and battery emissions from[7]

Subregion	Medium duty electric truck	Small electric van	Electric cargo bicycle	Quad-copter drone
AKGD	742.5	326.1	22.4	16.7
AKMS	838.1	367.6	24.9	18.7
AZNM	767.0	336.7	23.0	17.2
CAMX	592.2	260.8	18.4	13.5
ERCT	732.4	321.7	22.1	16.5
FRCC	594.4	261.7	18.5	13.5
HIMS	852.0	373.7	25.3	19.0
HIOA	954.6	418.3	28.0	21.2
MROE	893.5	391.7	26.4	19.9
MROW	981.7	430.0	28.7	21.8
NEWE	533.3	235.2	16.8	12.2
NWPP	905.6	397.0	26.7	20.2
NYCW	573.8	252.8	17.9	13.1
NYLI	713.4	313.4	21.6	16.1
NYUP	530.2	233.8	16.8	12.1
PRMS	912.8	400.1	26.9	20.3
RFCE	702.1	308.5	21.3	15.8
RFCM	940.5	412.1	27.6	20.9
RFCW	981.5	430.0	28.7	21.8
RMPA	904.2	396.3	26.7	20.1
SPNO	1059.1	463.6	30.8	23.5
SPSO	837.1	367.2	24.9	18.7
SRMV	655.1	288.1	20.1	14.8
SRMW	1009.1	441.9	29.5	22.4
SRSO	752.0	330.2	22.6	16.9
SRTV	837.2	367.2	24.9	18.7
SRVC	746.3	327.7	22.5	16.8
US	782.5	343.5	23.4	17.5

Table S4: Life cycle GHG emissions by vehicle per package for each EPA subregion [$gCO_2e/package$]. Direct emissions from EPA Subregions,[6] upstream electricity and battery emissions from,[7] and base-case delivery intensity as provided in S2

Subregion	Medium duty electric truck	Small electric van	Electric cargo bicycle	Quad-copter drone
AKGD	353.6	93.7	22.4	66.7
AKMS	399.1	105.6	24.9	74.9
AZNM	365.3	96.8	23.0	68.8
CAMX	282.0	74.9	18.4	53.8
ERCT	348.7	92.4	22.1	65.8
FRCC	283.0	75.2	18.5	54.0
HIMS	405.7	107.4	25.3	76.1
HIOA	454.6	120.2	28.0	84.9
MROE	425.5	112.6	26.4	79.6
MROW	467.5	123.6	28.7	87.2
NEWE	253.9	67.6	16.8	48.8
NWPP	431.3	114.1	26.7	80.7
NYCW	273.2	72.6	17.9	52.3
NYLI	339.7	90.1	21.6	64.2
NYUP	252.5	67.2	16.8	48.5
PRMS	434.7	115.0	26.9	81.3
RFCE	334.3	88.7	21.3	63.2
RFCM	447.9	118.4	27.6	83.7
RFCW	467.4	123.6	28.7	87.2
RMPA	430.6	113.9	26.7	80.6
SPNO	504.3	133.2	30.8	93.8
SPSO	398.6	105.5	24.9	74.8
SRMV	311.9	82.8	20.1	59.2
SRMW	480.5	127.0	29.5	89.5
SRSO	358.1	94.9	22.6	67.5
SRTV	398.7	105.5	24.9	74.8
SRVC	355.4	94.2	22.5	67.0
US	372.6	98.7	23.4	70.1

Supplemental References

[1] Rotaru, C. and Todorov, M. (2017). Helicopter Flight Physics. In Flight Physics - Models, Techniques and Technologies. InTech. 10.5772/intechopen.71516.

[2] Beard, R. W. and McLain, T. W. (2012). Small unmanned aircraft: Theory and practice. ISBN 9780691149219.

[3] DJI. MATRICE 100 SPECS. Available at `https://www.dji.com/matrice100/info`.

[4] Liu, Z., Sengupta, R., and Kurzhanskiy, A. (2017). A power consumption model for multi-rotor small unmanned aircraft systems. In 2017 International Conference on Unmanned Aircraft Systems (ICUAS), pages 310–315. IEEE. ISBN 978-1-5090-4495-5. 10.1109/ICUAS.2017.7991310.

[5] Baron, M. (2013). Probability and Statistics for Computer Scientists. In Probability and Statistics for Computer Scientists, chapter 11. Chapman and Hall/CRC, second edition edition. ISBN 9780429108761. 10.1201/B14800.

[6] US EPA (2020). Emissions & Generation Resource Integrated Database (eGRID).

[7] Laboratory, A. N. (2020). GREET.
